# Proteomic Analysis Unveils Expressional Changes in Cytoskeleton- and Synaptic Plasticity-Associated Proteins in Rat Brain Six Months after Withdrawal from Morphine

**DOI:** 10.3390/life11070683

**Published:** 2021-07-13

**Authors:** Zdenka Drastichova, Lucie Hejnova, Radka Moravcova, Jiri Novotny

**Affiliations:** Department of Physiology, Faculty of Science, Charles University, 12800 Prague, Czech Republic; zdenka.drastichova@natur.cuni.cz (Z.D.); lucie.hejnova@natur.cuni.cz (L.H.); radka.moravcova@natur.cuni.cz (R.M.)

**Keywords:** morphine, withdrawal, brain, proteomics, synaptic plasticity

## Abstract

Drug withdrawal is associated with abstinence symptoms including deficits in cognitive functions that may persist even after prolonged discontinuation of drug intake. Cognitive deficits are, at least partially, caused by alterations in synaptic plasticity but the precise molecular mechanisms have not yet been fully identified. In the present study, changes in proteomic and phosphoproteomic profiles of selected brain regions (cortex, hippocampus, striatum, and cerebellum) from rats abstaining for six months after cessation of chronic treatment with morphine were determined by label-free quantitative (LFQ) proteomic analysis. Interestingly, prolonged morphine withdrawal was found to be associated especially with alterations in protein phosphorylation and to a lesser extent in protein expression. Gene ontology (GO) term analysis revealed enrichment in biological processes related to synaptic plasticity, cytoskeleton organization, and GTPase activity. More specifically, significant changes were observed in proteins localized in synaptic vesicles (e.g., synapsin-1, SV2a, Rab3a), in the active zone of the presynaptic nerve terminal (e.g., Bassoon, Piccolo, Rims1), and in the postsynaptic density (e.g., cadherin 13, catenins, Arhgap35, Shank3, Arhgef7). Other differentially phosphorylated proteins were associated with microtubule dynamics (microtubule-associated proteins, Tppp, collapsin response mediator proteins) and the actin–spectrin network (e.g., spectrins, adducins, band 4.1-like protein 1). Taken together, a six-month morphine withdrawal was manifested by significant alterations in the phosphorylation of synaptic proteins. The altered phosphorylation patterns modulating the function of synaptic proteins may contribute to long-term neuroadaptations induced by drug use and withdrawal.

## 1. Introduction

Morphine is an opioid drug used as an effective analgesic for the treatment of postoperative and cancer pain. However, medication with morphine can create harmful side effects. Prolonged administration of this drug brings about a high risk of abuse and addiction, which manifests as physical dependence and/or psychological addiction [1]. Physical dependence stems from neuroadaptive changes which occur at the molecular and cellular levels in the central nervous system (CNS) and are associated with the appearance of withdrawal symptoms following the discontinuation of drug use [1,2,3]. In experimental conditions, the cessation of morphine use is accomplished by abruptly stopping chronic treatment with the drug or by administration of an opioid receptor antagonist which acts as a potent competitive inhibitor and blocks opioid receptors. In animals, this intervention evokes withdrawal symptoms such as jumping, paw tremors, teeth chattering, and diarrhea [1].

During drug administration and withdrawal, neuroadaptations occurring in the brain are associated with alterations in synapses, ion channels and structural compartments. In this respect, the greatest attention has been focused on neurotransmitter glutamatergic, dopaminergic and GABAergic systems [2,3,4]. However, it is highly likely that drug administration and withdrawal may induce changes in the expression and function of other proteins involved in the regulation of synaptic plasticity. The synapses, specialized sites capable of mediating communication between neurons in the CNS, are composed of pre- and postsynaptic compartments [5]. The presynaptic terminal contains the active zone (AZ) with proteins involved in the recruitment and exocytosis of synaptic vesicles (SVs), which release neurotransmitters into the synaptic cleft. The postsynaptic site contains the postsynaptic density (PSD) with receptors and signaling components that respond to neurotransmitters released from the presynaptic terminal [5]. Alterations in the expression and posttranslational modifications of synaptic proteins may cause synaptic dysfunction and subsequently lead to neurological disorders [5,6,7].

The long-lasting brain adaptations connected with compulsive drug use and craving are not limited to only one brain region. Although the ventral tegmental area (VTA) and the nucleus accumbens (NA) are widely recognized as brain regions critical for the development of drug addiction and reward [8,9], it was found that dopaminergic neurons from the VTA project not only to the NA but also to the dorsal striatum, prefrontal cortex and hippocampus and, conversely, the NA receives glutamatergic inputs from the cortex and hippocampus [10]. Dysregulated homeostasis in glutamatergic synapses is implicated in cognitive impairments and cravings associated with opioid use disorder [9]. The dopamine system is also involved in opioid use disorder because low dopamine D2/3 receptor availability and low presynaptic dopamine were found in the striatum of opioid dependent patients [11]. Addictive drugs also modify cerebellar glutamate and endocannabinoid interactions, norepinephrine and dopamine levels, and intracellular signaling transduction pathways [12]. The reduced release of dopamine in the NA may also be driven by increased activity of dynorphin and the κ-opioid receptor system in the ventral striatum, thereby contributing to the negative emotional state associated with withdrawal and protracted abstinence [13]. The hippocampus is critical for the formation of addictive memory and for triggering a relapse [14]. Drug-induced dysregulation of neuronal networks in the cortex contributes to the functional abnormalities of brain reward systems and causes compulsive drug use [13]. The cerebellum has been shown to be involved in emotional memory and experience, language, temporal perception, automatization of rules or decision making, which have all been found to be altered in addicted patients [12,15].

Morphine acts through opioid receptors (ORs). It has a high affinity for μ-OR and a somewhat lower affinity for κ- and δ-ORs [16]. The distribution of ORs differs between different regions of rat brain. The highest number of μ-OR is found in the striatum, a lower number in the hippocampus, and the lowest number in the cortex [17]. Although rat cerebellum was originally believed to be devoid of μ- and κ-ORs [18], some later studies reported opposite results; not only μ- and δ-ORs [19], but also κ-ORs [20] were found there. ORs are apparently less abundant in rat cerebellum when compared to other brain regions. Anyways, there are some indications that morphine can have a huge impact on cerebellar Purkinje cells [21]. Hence, the cerebellum should also be involved in dealing with opioid use disorder.

It has been observed that cytoskeletal dynamics may contribute to synaptic plasticity in addicted animals and the actin filaments were studied in the greatest detail in this respect [22]. The organization and remodeling of the microtubule network are also essential for synapse formation and stability. This network is affected by microtubule dynamic instability, branching, orientation, posttranslational modifications and interactions with microtubule-associated proteins (MAPs) [23,24]. Microtubules are composed of α- and β-tubulin heterodimers, whose posttranslational modifications might facilitate the recruitment of distinct MAPs for local regulation of microtubule function and are critical for neuronal health at all stages of life [24,25,26]. There are different types of MAPs with various functions and posttranslational modifications which affect their interaction with microtubules, microtubule dynamics and rearrangements of the microtubule network when neuroplasticity occurs [26]. Phosphorylation mediated by Ca^2+^/calmodulin-dependent kinase II (CaMKII) [26,27], glycogen synthase kinase III β (GSKIIIβ), cdc2 kinase, c-Jun N-terminal kinase 1 (JNK1), cAMP-dependent protein kinase (PKA), protein kinase C (PKC), and serine/threonine-protein kinase MARK [26] is an essential posttranslational modification of tubulins and MAPs. Tubulins and MAPs bind to many other interacting partners. Tubulin directly interacts with synapsin-1, a presynaptic vesicle protein, which is a neuronal phosphoprotein that contributes to the clustering of synaptic vesicles with cytoskeletal elements at the presynaptic terminals and thereby regulates SV cycling and neurotransmitter release [28]. Synapsin-1 can be phosphorylated at different phosphorylation sites by different kinases, and phosphorylation patterns coordinate the interaction of synapsin-1 with actin filaments or microtubules [28]. The other interacting partners of tubulins comprise tubulin polymerization-promoting protein (Tppp) [29] and stathmin [30]. The interacting partners of MAPs include calcium and potassium channels, neurotransmitter receptors, and spectrin [26,31]. Microtubules contribute to the formation, maintenance and function of axons and dendrites. Interestingly, modifications in dendrite branching as well as changed expression of tubulin, tau, stathmin and other cytoskeletal components were observed following chronic morphine treatment and spontaneous withdrawal [32,33].

Small GTPases of the Ras superfamily, which can be divided into several subfamilies such as Ras, Rho, Rab, Arf, and Ran, are important regulators of cytoskeletal reorganization. These GTP-binding proteins are involved in the regulation of cytoskeletal dynamics, vesicle trafficking and synaptic connectivity and plasticity [34,35,36,37,38,39]. Rab and Arf GTPases mediate coupling between microtubule motor proteins and vesicles, as well as transport of the cargo vesicles along microtubules and actin filaments to specific locations [35]. Rho GTPases play an essential role in changes of intracellular cytoskeleton dynamics [34,37]. Rab GTPases are known to modulate Rho activity to mediate cytoskeleton remodeling [35]. The activity of small GTPases is regulated by guanine nucleotide exchange factors (GEFs) and GTPase activator proteins (GAPs). While GEFs stimulate the exchange of GDP for GTP to activate small GTPases, GAPs promote GTP hydrolysis to inactivate them [34]. Most of the 150 GEFs and GAPs that have been identified so far are expressed in the brain with a specific spatial and temporal distribution pattern, but their function has not yet been elucidated [38]. Small GTPases and their regulators are modified by phosphorylation regulating their stability and activity, subcellular localization, and interactions with binding partners [40,41,42,43]. RhoA/Rho kinase signaling was shown to be downregulated in the NA of cocaine-dependent rats and may thus contribute to synaptic changes leading to drug addiction [44]. Rho signaling might also be related to synaptic plasticity in the amygdala and prefrontal cortex, which were identified as regulators of the reward circuitry as well [34].

The present study aimed to explore the long-term impact of morphine withdrawal on proteomic profiling of selected rat brain regions. It is known that drug addiction is associated with dysregulation of the dopamine and glutamate systems [9]. However, it would be too simplistic to assume that this process is mediated only by changes in neurotransmitters and their receptors. It is imaginable that dysregulation of neurotransmitter systems is associated with changes in the synaptic vesicle cycle that is comprised of several individual processes. It is also imaginable that postsynaptic signal transmission depends not only on the number of receptors on the postsynaptic membrane but also on the composition of the postsynaptic density. Likewise, changes in neurotransmitter signaling, which may affect cognitive functions (e.g., memory and learning, addiction, reward, motivation, and habits), are presumably related to changes in protein expression and protein posttranslational modifications determining the protein functions. The comprehensive analysis of the proteome and phosphoproteome can help us determine which of the components of synaptic vesicles, proteins of the presynaptic active zone and the postsynaptic density are affected by morphine, and reveal which synaptic processes could potentially be modulated by pharmacotherapeutic interventions during the withdrawal period.

## 2. Materials and Methods

### 2.1. Materials

Morphine sulfate was obtained from Saneca Pharmaceutical, Ltd., (Hlohovec, Slovakia) and BCA assay kit was from Thermo Fisher Scientific Inc. (Carlsbad, CA, USA). All other chemicals were purchased from Sigma-Aldrich (St. Louis, MO, USA) and were of the highest purity available.

### 2.2. Animals, Morphine Treatment and Withdrawal

Male Wistar rats (approximately 8 weeks of age) were purchased from Velaz, Ltd., Prague, Czech Republic. Rats were housed in groups of 3/cage in standard plastic cages containing wood chip bedding. They were maintained at normal ambient temperature (22 ± 1 °C) under a stable light-dark cycle (12 h light and 12 h darkness), and were allowed free access to food and water. All procedures were performed according to national and institutional guidelines for the care and use of animals in laboratory research. The protocols were approved by the Ministry of Education, Youth and Sports of the Czech Republic (license no. MSMT-1479/2019–6). Repeated administration of morphine was performed according to previously established procedures [45,46]. Rats were injected with increasing doses (10–50 mg/kg per day) of morphine dissolved in 0.9% NaCl for 10 consecutive days. Control animals received 0.9% NaCl. After cessation of morphine administration, and throughout the following 6 months of abstinence, rats were kept under standard housing conditions with food and water ad libitum.

### 2.3. Brain Tissue Homogenization and Digestion

The brains from morphine-treated (MOR; n = 9) and control (CON; n = 9) rats were rapidly removed, dissected and snap frozen in liquid nitrogen and stored at −80 °C until use. In order to obtain a reasonable number of samples for proteomic analyses and, simultaneously, to preserve the effect of the intrinsic biological variation, three pooled samples of prefrontal cortex (Ctx), hippocampus (Hp), striatum (Str) or cerebellum (Cb) were prepared for both MOR and CON groups by mixing equal amounts of respective brain tissues from three trios of randomly selected animals in each group. The pooled brain tissue samples were homogenized in 10 volumes of TMES buffer (20 mM Tris, 3 mM MgCl_2_, 1 mM EDTA, 250 mM sucrose; pH 7.4) containing protease and phosphatase inhibitors (cOmplete and PhosSTOP) using a glass-Teflon homogenizer (1200 rpm, 10 strokes), mixed 1:1 with 2% SDC (sodium deoxycholate) in 100 mM TEAB (triethylammonium bicarbonate; pH 8.0), and sonicated for 3 × 10 s in 2.0 mL Eppendorf tubes using a Bandelin UW 2070 sonicator (40% amplitude), as described previously [46]. Samples were cleared (14,000× *g*, 10 min). Protein concentration was determined using the Pierce BCA protein assay kit with bovine serum albumin as calibration standard and 250 µg of protein per sample were used for MS sample preparation. Proteins were digested by 5 µg of trypsin per sample at 37 °C overnight. Phosphopeptides were enriched using TiO_2_ according to [47].

### 2.4. nLC-MS2 Analysis

Nano reversed phase columns (EASY-Spray column, 50 cm × 75 µm ID, PepMap C18, 2 µm particles, 100 Å pore size) were used for LC/MS analysis. Mobile phase buffer A was composed of water and 0.1% formic acid. Mobile phase B was composed of acetonitrile and 0.1% formic acid. Samples were loaded onto the trap column (C18 PepMap100, 5 μm particle size, 300 μm × 5 mm, Thermo Scientific) for 4 min at 18 μL/min loading buffer was composed of water, 2% acetonitrile and 0.1% trifluoroacetic acid. Peptides were eluted with Mobile phase B gradient from 2% to 35% B in 60 min. Eluting peptide cations were converted to gas-phase ions by electrospray ionization and analyzed on a Thermo Orbitrap Fusion (Q-OT- qIT, Thermo Fisher Scientific (Cleveland, OH, USA). Survey scans of peptide precursors from 350 to 1400 m/z were performed in orbitrap at 120K resolution (at 200 *m*/*z*) with a 1 × 10^6^ ion count target. Tandem MS was performed by isolation at 1.5 Th with the quadrupole, HCD fragmentation with normalized collision energy of 35, and rapid scan MS analysis in the ion trap. The MS2 ion count target was set to 104 and the max injection time was 150 ms. Only those precursors with charge state 2–6 were sampled for MS2. The dynamic exclusion duration was set to 30 s with a 10 ppm tolerance around the selected precursor and its isotopes. Monoisotopic precursor selection was turned on. Cycle time was set to 2 s.

### 2.5. Data Analysis

All data were analyzed and quantified with the MaxQuant software (version 1.6.3.4, Planck Institute of Biochemistry, Munich, Germany) [48]. The false discovery rate (FDR) was set to 1% for both proteins and peptides and we specified a minimum peptide length of seven amino acids. The Andromeda search engine was used for the MS/MS spectra search against the Rattus norvegicus database (Uniprot, https://www.uniprot.org; assessd on 18 August 2020) containing 29,958 entries. Enzyme specificity was set as C-terminal to Arg and Lys, also allowing cleavage at proline bonds and a maximum of two missed cleavages. Carbamidomethylation of cysteine was selected as fixed modification and N-terminal protein acetylation and methionine oxidation as variable modifications. The “match between runs” feature of MaxQuant was used to transfer identifications to other LC-MS/MS runs based on their masses and retention time (maximum deviation 0.7 min) and this was also used in quantification experiments. Quantifications were performed with the label-free algorithm in MaxQuant [49]. Data analysis was performed using Perseus 1.6.1.3 software [50]. For quantification, intensities were determined as the intensity maximum over the retention time profile. To evaluate the degree of uniqueness, unique plus razor peptides were included for quantification. Only those phosphosites with localization probability higher than 0.75 were used for further data analysis.

Gene Ontology (GO) enrichment analysis of proteomic and phosphoproteomic profiles for each brain region was performed using appropriate online annotation tools (ShinyGO v0.61 and gProfiler); the p-value threshold was set to 10^−5^ for biological processes.

## 3. Results

### 3.1. GO Enrichment Analysis of Differentially Phosphorylated Proteins after a 6-Month Morphine Withdrawal

In order to evaluate the differences in phosphoproteomes of selected brain regions from control and morphine-withdrawn rats, bioinformatics analysis of data acquired by bottom-up label-free LC-MS proteomics was conducted using the MaxQuant and Perseus software platforms. A qualitative change was defined as the absence/presence of a protein in one experimental group in pairwise comparison under the condition that the protein was detectable/undetectable at least in two of three biological replicates. A quantitative change was defined as an at least two-fold difference in protein expression level between two experimental groups and concurrently the protein was detected at least in two of three biological replicates. By comparison of control and morphine-withdrawn rats, we identified 185 differentially phosphorylated sites on 109 phosphoproteins in cortex, 454 differentially phosphorylated sites on 256 phosphoproteins in hippocampus, 313 differentially phosphorylated sites on 174 phosphoproteins in striatum and 288 differentially phosphorylated sites on 183 phosphoproteins in cerebellum. The sets of altered phosphoproteins for each brain region were examined by GO enrichment analysis using the gProfiler tool (https://biit.cs.ut.ee/gprofiler_beta/gost; assessed on 12 February 2021) in order to specify biological processes which were affected by a six-month morphine withdrawal. A list of enriched GO terms related to synaptic plasticity, cytoskeleton organization and regulation of GTPase activity for each brain region (Table 1) includes p-values and numbers of differentially phosphorylated proteins. The detailed data of these GO enriched terms are stated in Table S1, including GO term IDs, negative logarithms of p-values, sizes of GO terms, numbers and gene names of altered proteins associated with GO terms.

The GO enriched terms related to synaptic plasticity (e.g., synaptic vesicle cycle, signal release from the synapse, neurotransmitter secretion and transport) and cytoskeleton organization were found in the cortex (Table 1 and Supplementary Table S1) but no altered proteins were found in biological processes followed in this study. The GO terms related to synaptic plasticity, cytoskeleton organization and regulation of GTPase activity were found in the hippocampus and striatum (Table 1 and Supplementary Table S1). In the hippocampus, synaptic processes affected by long-term morphine withdrawal included synaptic signaling, synaptic vesicle cycle, synapse organization and neurotransmitter secretion and transport, actin filament-based processes and microtubule cytoskeleton organization involved in mitosis (Table 1). The altered phosphoproteins Arhgef7, Nf1 and Rgs14 are involved in modulation of synaptic plasticity, cytoskeleton organization and regulation of GTPase activity. The altered phosphoproteins Add2, Mapt, Myh10, Palm and Ppfia1 are associated with cytoskeletal and synaptic processes, proteins Arhgap35 and Stmn3 with cytoskeletal processes and regulation of GTPase activity and proteins Rasgrf1, Sema4d, Stxbp5l and Syngap1 with synaptic processes and regulation of GTPase activity (Supplementary Table S1). In the striatum, similar GO enriched terms were included in the list as in the hippocampus, but the number of GO enriched terms was smaller (Table 1). Only four proteins (Arhgap35, Bcr, Crk and Hdac6) establish associations between synaptic plasticity, cytoskeleton organization and regulation of GTPase activity according to GO enrichment analysis (Supplementary Table S1). In the cerebellum, GO enriched terms were related to synaptic plasticity and similar to those found in the cortex, hippocampus and striatum (Table 1). The most GO enriched terms related to synaptic plasticity, cytoskeleton organization and regulation of GTPase activity were found in the hippocampus, where the largest set of altered phosphoproteins was determined. Moreover, the most extensive relations were observed in this brain region, given that several phosphoproteins were involved at least in two of three biological processes examined in this study.

When comparing the appearance of differentially phosphorylated proteins involved in single biological processes, the greatest similarity between different brain regions was noticed for cytoskeleton organization. Microtubule-associated proteins Map1a, Map1b, Map2 and Mapt, erythrocyte membrane proteins Epb41l1 and Epb41l3, as well as other proteins such as Add2, Cdc42bpb, Dpysl3, Marcks and Palm were found in the list of GO enriched terms related to cytoskeleton organization in all four brain regions (Supplementary Table S1). Regarding synaptic biological processes, phosphoproteins Syn1 and Bsn were identified in all four brain regions (Supplementary Table S1).

### 3.2. GO Enrichment Analysis of Differentially Expressed Proteins after a 6-Month Morphine Withdrawal

In order to evaluate the differences in proteomes of selected brain regions from control and morphine-withdrawn rats, bioinformatic analysis of data acquired by bottom-up label-free LC-MS proteomics was conducted using the MaxQuant and Perseus software platforms. A qualitative change was defined as the absence/presence of a protein in one experimental group in pairwise comparison under the condition that the protein was un/detectable at least in two of three biological replicates. A quantitative change was defined as an at least two-fold difference in protein expression level between two experimental groups and concurrently the protein was detected at least in two of three biological replicates. The levels of 79, 51, 78 and 175 proteins were altered by long-term morphine withdrawal in the cortex, hippocampus, striatum and cerebellum, respectively. The sets of differentially expressed proteins for each brain region were assessed by GO enrichment analysis using the ShinyGO v0.61 tool (http://bioinformatics.sdstate.edu/go/; assessed on 9 March 2021) in order to specify biological processes which were affected by a six-month morphine withdrawal. A list of enriched GO terms related to synaptic plasticity, cytoskeleton organization and regulation of GTPase activity for each brain region (Table 1) includes FDR values and numbers of altered proteins. The detailed data of these GO enriched terms are stated in Table S2, including gene names and numbers of altered proteins associated with GO terms.

The most enriched GO terms inferred from the differences in protein expression in cortex were related to synaptic plasticity and signaling, synapse assembly and neurotransmitter receptor transport (Table 1 and Supplementary Table S2). Two GO terms related to synaptic plasticity were found in the hippocampus. While GO enriched terms identified in the striatum were related to cytoskeleton organization and actin filament-based process, those in the cerebellum were linked to signal transduction mediated by small GTPases (Table 1 and Supplementary Table S2).

Using GO enrichment analysis, no association between synaptic plasticity, cytoskeleton organization and regulation of small GTPase activity was found in the proteomic profiles of differentially expressed proteins. Biological processes related to synaptic plasticity were identified in the cortex and hippocampus, biological processes related to cytoskeleton organization in striatum and biological processes related to the regulation of small GTPase activity in the cerebellum. The numbers of differentially expressed proteins in the cortex, hippocampus and striatum were smaller than the numbers of differentially phosphorylated proteins, which might decrease the chance of finding associations between the proteins altered by morphine withdrawal.

### 3.3. Changes in Protein Expression and Phosphorylation Induced by a 6-Month Morphine Withdrawal

The datasets of differentially phosphorylated proteins associated with synaptic plasticity, cytoskeleton organization and regulation of GTPase activity consist of 78 phosphosites from 42 phosphoproteins in cortex, 188 phosphosites from 95 phosphoproteins in hippocampus, 113 phosphosites from 59 phosphoproteins in striatum, and 96 phosphosites from 49 phosphoproteins in cerebellum (Supplementary Table S3). Long-term morphine withdrawal elicited distinct changes in the phosphorylation of different phosphosites, resulting in the formation of unique phosphorylation profiles in different brain regions. For better clarity and transparency, changes in phosphorylation of some phosphoproteins are listed separately for the cortex (Table 2), hippocampus (Table 3), striatum (Table 4), and cerebellum (Table 5).

**Figure 1:**
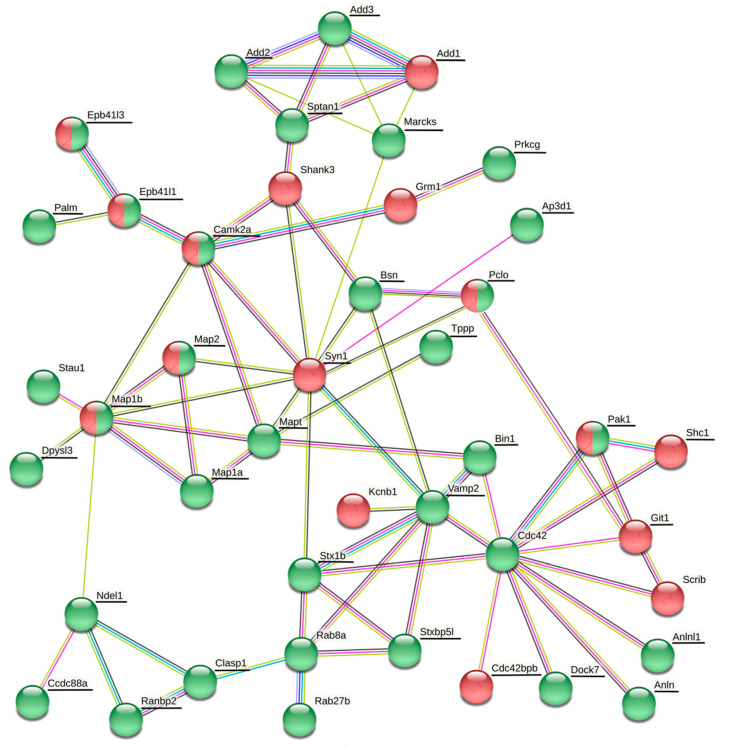
Network representation of differentially expressed or phosphorylated proteins in rat cortex six months after morphine withdrawal. Forty-four differentially expressed or phosphorylated proteins were mapped onto the rat String database of protein-protein associations and connected in a tight network with 71 edges. The green and red nodes represent proteins with increased and decreased levels, respectively, in the cortex of morphine-withdrawn rats relative to controls. The names of differentially phosphorylated proteins are underlined. Edges between the nodes represent the connection or relationship between the respective proteins. The average node degree is 3.23 and enrichment *p*-value of protein–protein associations is <1.0e−16.

**Figure 2:**
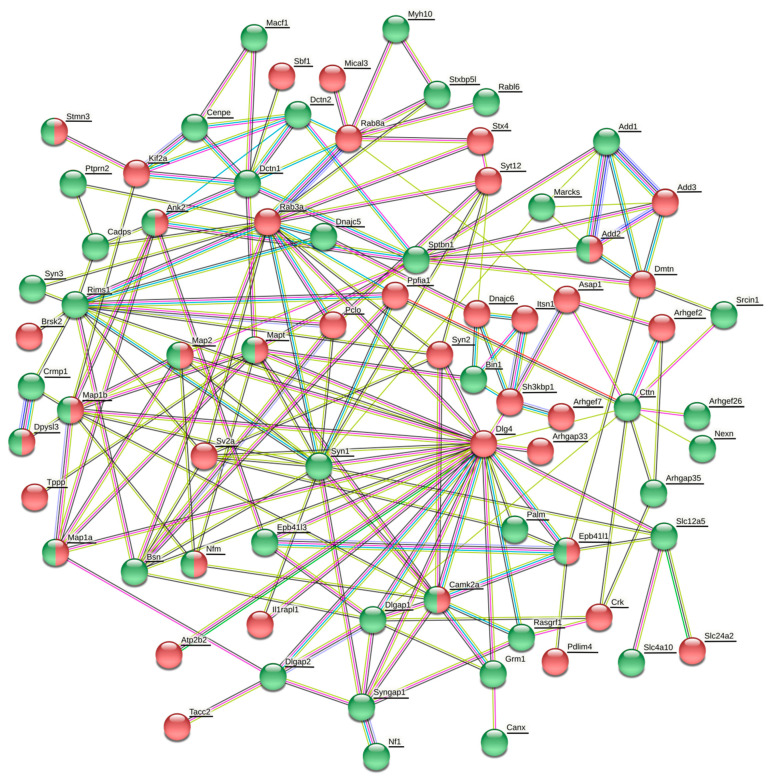
Network representation of differentially expressed or phosphorylated proteins in rat hippocampus six months after morphine withdrawal. Seventy-five differentially expressed or phosphorylated proteins were mapped onto the rat String database of protein–protein associations and connected in a tight network with 183 edges. The green and red nodes represent proteins with increased and decreased levels, respectively, in the hippocampus of morphine-withdrawn rats relative to controls. The names of differentially phosphorylated proteins are underlined. Edges between the nodes represent the connection or relationship between the respective proteins. The average node degree is 4.88 and enrichment *p*-value of protein–protein associations is <1.0e−16.

**Figure 3:**
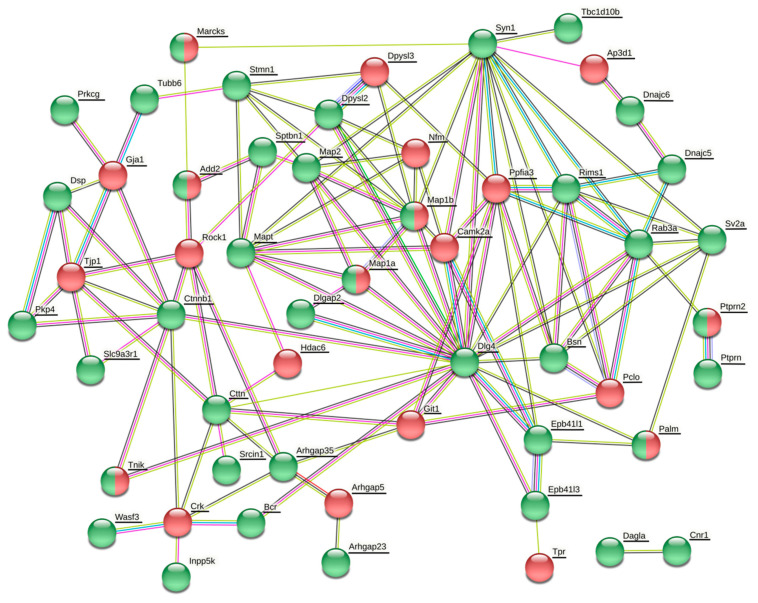
Network representation of differentially expressed or phosphorylated proteins in rat striatum six months after morphine withdrawal. Fifty-four differentially expressed or phosphorylated proteins were mapped onto the rat String database of protein–protein associations and connected in a tight network with 124 edges. The green and red nodes represent proteins with increased and decreased levels, respectively, in the striatum of morphine-withdrawn rats relative to controls. The names of differentially phosphorylated proteins are underlined. Edges between the nodes represent the connection or relationship between the respective proteins. The average node degree is 4.59 and enrichment *p*-value of protein–protein associations is <1.0e−16.

**Figure 4:**
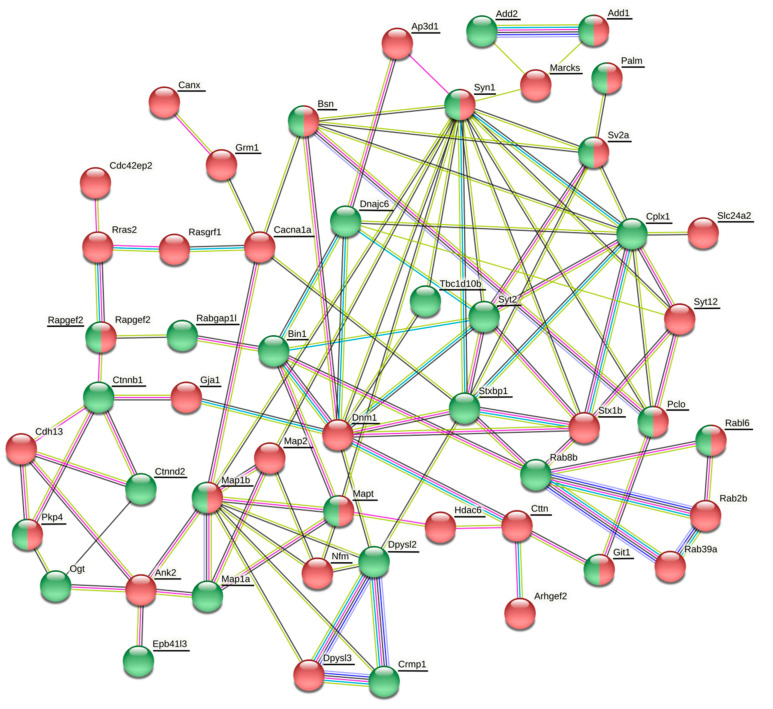
Network representation of differentially expressed or phosphorylated proteins in rat cerebellum six months after morphine withdrawal. Fifty-one differentially expressed or phosphorylated proteins were mapped onto the rat String database of protein–protein associations and connected in a tight network with 107 edges. The green and red nodes represent proteins with increased and decreased levels, respectively, in the striatum of morphine-withdrawn rats relative to controls. The names of differentially phosphorylated proteins are underlined. Edges between the nodes represent the connection or relationship between the respective proteins. The average node degree is 4.20 and enrichment *p*-value of protein–protein associations is <1.0e−16.

The datasets of differentially expressed proteins associated with synaptic plasticity, cytoskeletal organization and GTPase regulatory activity comprise 9 proteins in the cortex, 4 proteins in the hippocampus, 8 proteins in the striatum and 10 proteins in the cerebellum (Table 7). The details, including quantification and statistics, QC (quantification control) parameters of identification and samples data binary logarithms, are stated in Supplementary Table S4. In order to determine the associations between differentially phosphorylated and expressed proteins, protein–protein association networks were created for each brain region using the String database (https://string-db.org/; assessed on 22 March 2021) with color depiction of alterations in protein levels (Figure 1, Figure 2, Figure 3 and Figure 4).

We observed alterations in phosphorylation of synapsin-1, Bassoon and Piccolo proteins, regulating synaptic membrane exocytosis protein 1 (Rims1), Rab3a, synaptic vesicle glycoprotein SV2A, synaptotagmin-2, syntaxin-1b, syntaxin-binding protein 5-like (Stxbl5l), Bin1 protein, Ppfia proteins, cytoplasmic linker-associated protein 1 (Clasp), MAP1B, Tau, Srcin1, Akap12, Tnik, diydropyrimidinase-related proteins (Dpysl proteins and Crmp1), serine/threonine-protein kinase MRCK beta (Cdc42bpb), serine/threonine-protein kinase Brsk2 and DnaJ homolog 6 (Dnajc6) (Table 2, Table 3, Table 4 and Table 5 and Supplementary Table S3), which are either components of synaptic vesicles or participate in synaptic vesicle transport and exocytosis in the active zone [51,52,53,54]. In the cortex, we found that a six-month morphine withdrawal increased the level of Rab27b (Table 6), which participates in synaptic vesicle exocytosis and recycling at the presynaptic nerve terminal [55]. Another group of differentially phosphorylated synaptic phosphoproteins included MAP1A, MAP1B, MAP2, tau, Camk2a, Tppp (brain specific protein p25 alpha), SynGAP1, δ-catenin, spectrin alpha chain, spectrin beta chain, cortactin, ankyrin 2, paralemmin-1, band 4.1-like protein 1, band 4.1-like protein 3, calnexin and RGS14 (Table 2, Table 3, Table 4 and Table 5). The levels of Shank3 and cadherin 13 were found to be decreased after a six-month morphine withdrawal in cortex and cerebellum, respectively (Table 6). All these proteins represent components of postsynaptic multimeric complexes [51,54,56,57]. The third group differentially phosphorylated or expressed proteins included Cdc42 and regulators of Rho/Cdc42 GTPases, Rho-associated protein kinase 1 (Rock1), Rab proteins and its regulator RabGAP1l, Rras2, and regulators of Ras/Rap GTPases (Table 2, Table 3, Table 4, Table 5 and Table 6, Supplementary Tables S3 and S4). While many Rab GTPases are located in the presynaptic nerve terminal and are involved in biogenesis, transport, docking, exocytosis and recycling of synaptic vesicles [55], Rab8 proteins are involved in GluA1-AMPA receptors trafficking from the endoplasmatic reticulum to the Golgi complex and in their delivery to the postsynaptic membrane [58]. Ras and Rap GTPases contribute to linking NMDA receptor activation and calcium influx with phosphorylation and trafficking of AMPA receptors during the induction of synaptic plasticity [59]. Rho and Cdc42 GTPases are located at the presynaptic nerve terminals as well as in the postsynaptic cells. Cdc42 signaling can induce actin cytoskeleton remodeling and subsequently trafficking or scaffolding of vesicles or key exocytosis molecules to the presynaptic nerve terminal [60]. Cdc42 signaling and RhoA/Rock signaling are both located in the postsynaptic neurons [56].

#### 3.3.1. Changes in Protein Expression and Phosphorylation in the Cortex

Proteins related to synaptic vesicle arrangement and cycling in cortex were mostly hyperphosphorylated after a six-month morphine withdrawal (Figure 1 and Table 2). Synapsin-1 together with Cdc42bpb and Srcin1 were found to be hypophosphorylated. Piccolo and MAP1B were simultaneously hyper- and hypophosphorylated at different phosphosites. Some other proteins (Bsn, Stx1b, Stxbl5l, Bin1, Clasp, tau, Dpysl3) were hyperphosphorylated. This was accompanied by an increase in the level of Rab27b (Table 6), which is together with Rab3 engaged in synaptic vesicle docking and exocytosis [58]. Likewise, proteins forming postsynaptic complexes were mostly hyperphosphorylated (Figure 1 and Table 2). MAP1A, tau, TPPP, δ-catenin, spectrin α-chain, paralemmin-1, band 4.1-like protein 1 and band 4.1-like protein 3 were hyperphosphorylated and only Git1 was hypophosphorylated. Three proteins (MAP1B, MAP and Camk2a) were simultaneously hyper- and hypophosphorylated. This was accompanied by a decrease in the level of Shank3 (Table 6), which colocalizes with Bassoon in the presynaptic nerve terminal [61], and forms multimeric sheets within the postsynaptic density where they interact with numerous PSD proteins and the actin cytoskeleton [62]. Scribble planar cell polarity protein (Scrib), which acts a scaffold for the recruitment of proteins and directs membrane localization of SVs [63], was downregulated (Table 6). The level of Cdc42 homolog was increased in cortex from morphine-withdrawn rats (Table 6) and Cdc42bpb (MRCK beta kinase) was hyperphosphorylated at Ser1695 phosphosite located in the C-terminus of the protein (Table 2).

#### 3.3.2. Changes in Protein Expression and Phosphorylation in the Hippocampus

There was no clear trend in the level of phosphorylation of proteins related to synaptic vesicle arrangement and cycling in the hippocampus after a six-month morphine withdrawal (Figure 2 and Table 3). Synapsin-1 together with Bassoon, Rims1, Stxbl5l, Bin1, Srcin1, Akap12, Crmp1 and Dnajc5 were hyperphosphorylated (Table 3). Piccolo, Rab3a, SV2a protein, Ppfia1, Cdc42bpb, Brsk2 and Dnajc6 were hypophosphorylated. MAP1B and tau proteins with Dpysl3 were found to be simultaneously hyperphosphorylated at some phosphosites and hypophosphorylated at others (Figure 2 and Table 3). Likewise, proteins forming postsynaptic complexes were mostly hyper- and hypophosphorylated to the same extent (Figure 2 and Table 3). MAPs (MAP1A, MAP1B, MAP2 and tau) together with Camk2a, Ank2, band 4.1-like protein 1 were simultaneously hyper- and hypophosphorylated at different phosphosites. SynGAP1, δ-catenin, spectrin β chain, cortactin, paralemmin-1, band 4.1-like protein 3, calnexin and Dlgap2 were hyperphosphorylated and Tppp, Rgs14 and Dlg4 were hypophosphorylated (Figure 2 and Table 3). The observed changes in phosphoproteome were accompanied by a downregulation of interleukin-1 receptor accessory protein-like 1 (Il1rapl1) (Table 6), which promotes formation of excitatory synapses [64]. Small GTPases were not affected by a six-month morphine withdrawal, except for changed phosphorylation of Rab3a and Rabl6 (Table 3). On the other hand, the level of phosphorylation of regulators of Rho/Cdc42 GTPases, Arhgap and Arhgef, was markedly changed. Arhgap33 was hypophosphorylated at Thr974 and Arhgap35 was hyperphosphorylated at Ser1179 (Table 3). The Ser1179 phosphosite at Arhgap35 is located in the p120RasGAP binding domain [43]. Three Arhgef proteins were differentially phosphorylated (Table 3). Whereas Arhgef26 was hyperphosphorylated, Arhgef7 (β-PIX) was hyperphosphorylated at Ser228 and Arhgef2 (Lfc) at Ser916 (Table 3). Ral and Ran GTPase binding proteins, Ralbp1 and Ranbp3, were hypophosphorylated and hyperphosphorylated, respectively. Rasgrf1 was hyperphosphorylated at Ser747 (Table 3). These data suggest that a six-month morphine withdrawal exerts a great impact on GTPase signaling, mainly on Rho/Cdc42 signaling via its GAP and GEF regulators and on Ras signaling via its releasing factor Rasgrf1 and Ras/Rap GTPase activating protein SynGAP1, and indirectly via phosphorylation of Arhgap35 in its p120RasGAP binding domain.

#### 3.3.3. Changes in Protein Expression and Phosphorylation in the Striatum

Proteins related to synaptic vesicle arrangement and cycling were mostly hyperphosphorylated after a six-month morphine abstinence (Figure 3 and Table 4). Synapsin-1 together with Cdc42bpb, Srcin1, Bassoon, Rims1, Rab3a, SV2a, Tau, Dpysl2 and Dnajc5 were found to be hyperphosphorylated. Piccolo, Ppfia3 and Dpysl3 were hypophosphorylated. MAP1B and Tnik were simultaneously hyper- and hypophosphorylated at different phosphosites. Likewise, proteins forming postsynaptic complexes were mostly hyperphosphorylated (Figure 3 and Table 4). MAP2, tau, β-catenin, spectrin β-chain, cortactin, band 4.1-like protein 1, band 4.1-like protein 3 were hyperphosphorylated and only Git1 and Camk2a were hypophosphorylated. MAP1A, MAP1B and paralemmin-1 were simultaneously hyper- and hypophosphorylated (Figure 3 and Table 4). The β-tubulin isoform Tubb6, belonging to the building blocks of microtubules, was upregulated (Table 6). Long-term morphine withdrawal diversely affected proteins implicated in signaling mediated by small G proteins. Contrary to hyperphosphoryled Rab3a, the other Rab protein, Rabl6, was hypophosphorylated. Interestingly, Arhgap proteins were distinctly phosphorylated (Table 4). Whereas Arhgap23 and Arhgap35 (p190RhoGAP) were hyperphosphorylated, Arhgap31 (Cdc42GAP) and Arhgap5 were hypophosphorylated. Arhgap35 was hyperphosphorylated at Tyr1105; this residue is phosphorylated by Src kinase and is involved in the association of Arhgap35 with p120RasGAP [43]. These changes were accompanied by a decrease in expression of Rock1, Rho-associated protein kinase 1 associated with cadherin/catenin or cortactin/Srcin1 complexes (Figure 2).

#### 3.3.4. Changes in Protein Expression and Phosphorylation in the Cerebellum

There was no clear trend in the level of phosphorylation of proteins related to synaptic vesicle arrangement and cycling in the cerebellum after a six-month morphine withdrawal (Figure 4 and Table 5). Synapsin-1 together with Bassoon, Piccolo, SV2a protein, MAP1B and tau were found to be simultaneously hyperphosphorylated at some phosphosites and hypophosphorylated at others. While the Stx1b, Akap12 and Dpysl3 proteins were only hypophosphorylated, synaptotagmin-2, Bin-1, Dpysl2, Crmp1 and Dnajc 6 were only hyperphosphorylated. Interestingly, phosphorylation of synaptotagmin-2 at Thr125 increased nearly three hundred times (Table 5). Likewise, proteins forming postsynaptic complexes were mostly hyper- and hypophosphorylated to the same extent (Figure 4 and Table 5). MAP1B, tau, paralemmin-1 and Git1 were simultaneously hyper- and hypophosphorylated at different phosphosites. Whereas MAP1A, β- and δ-catenins and band 4.1-like protein 3 were hyperphosphorylated, MAP2, cortactin and calnexin were hypophosphorylated (Figure 4 and Table 5). This was accompanied by a decrease in the expression of cadherin 13 (Table 6). Small GTPases and their regulators were altered mainly at the level of protein expression than phosphorylation. Rab2b, Rab39 and Rras2 were downregulated and Rab8b was upregulated (Table 6). The Rabl6 protein was simultaneously hyper- and hypophosphorylated at different phosphosites (Table 5). The expression levels of four GTPase regulators (Arhgef2, Cdc42ep2, Rapgef2 and Rasgrf1) were decreased (Table 6) suggesting regulation of several GTPase signaling pathways. Interestingly, Rapgef2 and Rabgap1l were simultaneously hyperphosphorylated at Ser1115 and at Ser490, respectively (Table 5).

#### 3.3.5. Comparison of Changes in Pre- and Postsynaptic Protein Clusters between Different Brain Regions

To determine which presynaptic and postsynaptic multimeric complexes were most affected by a six-month morphine withdrawal, some altered proteins were arranged into seven clusters according to their function or interaction with each other (Table 7). Three protein clusters involving components of synaptic vesicles, synaptic vesicle exocytosis and components of the active zone represent multimeric protein complexes located in the presynaptic nerve terminal. The clusters of proteins involved in the cadherin–catenin complex or in a scaffold structure of the postsynaptic density are located in dendritic spines. Two clusters of proteins were associated with the actin–spectrin network or microtubule dynamics. From presynaptic protein clusters, proteins of the active zone were apparently most affected because alterations in protein phosphorylation frequently occurred in all the four brain regions under scrutiny. The phosphoproteins present in synaptic vesicles were also differentially phosphorylated in all four brain regions. Interestingly, there were fewer changes in the cortex compared with the other three regions. Synaptic vesicle exocytosis was affected only in the cortex and cerebellum, and the observed alterations differed markedly between these two regions.

In general, the cadherin–catenin complex was affected by a six-month morphine withdrawal and changes occurred mainly on the level of catenin phosphorylation in all four brain regions, but some regional differences were observed (Table 7). First, no other protein involved in the cadherin–catenin complex was found to be altered in the cortex. Second, the RhoA signaling pathway was greatly affected in the striatum, where Arhgap35, a negative regulator of RhoA, was phosphorylated at Tyr1105 (Table 4). Importantly, Tyr1105 phosphorylation was shown to be required for RhoA inactivation [43]. The expression of Rock1 kinase, which is activated by RhoA, was decreased. These data indicate that RhoA signaling pathway is suppressed in the striatum after a six-month morphine withdrawal. Third, alteration in the cadherin family was found only in the cerebellum, where the level of cadherin 13 was decreased.

The level of Shank3, a scaffold protein localized in the postsynaptic density, which acts as a modulator of small GTPases and actin dynamics, was decreased in the cortex but not changed in the other three brain regions where its interacting protein, cortactin, was differentially phosphorylated (Table 7). In hippocampus, the Shank3 interacting partner, Arhgef7 (β-PIX), was differentially phosphorylated at Ser228 (Table 3) localized in the Dbl domain outside the domains mediating interaction with Shank3 [65], indicating that the interaction of Shank3 and Arhgef7 was not affected by a six-month morphine withdrawal. The Dbl domain is associated with activation of Rho GTPases [66], suggesting yet another possibility how the Rho signaling pathway could be affected. Shank3 protein and its interacting proteins affect the actin cytoskeleton via formation and stabilization of F actin and subsequently spine morphogenesis via enhancement of spine maturation [67]. Thus, a six-month morphine withdrawal could differently affect spine morphogenesis in all four brain regions under investigation.

Spectrins, ankyrin 2 and adducins, which were found to be differentially phosphorylated (Table 7), together with actin filaments form a membrane-associated periodic skeleton (MPS). They are ubiquitously distributed in mature axons, where F-actin rings are arranged periodically by spectrin tetramer spacers [68]. Adducins and band 4.1-like protein 1 stabilize interaction between actin and spectrin [68], suggesting regional changes in actin-spectrin interactions induced by a six-month morphine withdrawal.

The microtubule-associated proteins belong to a few proteins which were differentially phosphorylated in all four brain regions (Table 7). As a rule, these proteins were altered at several different phosphosites (Table 2, Table 3, Table 4 and Table 5). MAP1B, which is localized in the presynaptic nerve terminal as well in postsynaptic cells, is a microtubule-associated protein with the largest number of altered phosphosites in all four brain regions (Table 2, Table 3, Table 4 and Table 5). Dihydropyriminase-related proteins Crmp1 and Dpysl2 were found to be differentially phosphorylated in the hippocampus, striatum and cerebellum (Table 7), suggesting that microtubule stability was more affected in these brain three regions than in the cortex.

**Table 7:** Comparison of changes induced by a six-month morphine withdrawal in protein clusters comprising presynaptic or postsynaptic multimeric complexes in selected brain regions.

Protein	Function	Alteration	Ctx	H	S	Cb
Synapsin-1	Components of synaptic vesicles [69]	Phosphorylation	↓	↑	↑	↑↓
SV2a	Components of synaptic vesicles [69]	Phosphorylation	-	↓	↑	↑↓
Synaptotagmin-2	Components of synaptic vesicles [69]	Phosphorylation	-	-	-	↑
Rab3a	Components of synaptic vesicles [69]	Phosphorylation	-	↓	↑	-
Rab27b	Synaptic vesicle exocytosis [55]	Expression	↑	-	-	-
Syntaxin-1B	Component of SNARE complex [70]	Phosphorylation	↑	-	-	↓
Complexin-1	Interaction with syntaxin-1 [70]	Phosphorylation	-	-	-	↑
Stxbp1 (Munc18-1)	Interaction with syntaxin-1 [70]	Phosphorylation	-	-	-	↑
Vamp2 (Synaptobrevin)	Interaction with syntaxin-1 [70]	Phosphorylation	↑	-	-	-
Bassoon	Protein of active zone [71]	Phosphorylation	↑	↑	↑	↑↓
Piccolo	Protein of active zone [71]	Phosphorylation	↑↓	↓	↓	↑↓
Ppfia3 (Liprin α3)	Protein of active zone [71]	Phosphorylation	-	-	↓	-
Rims1	Protein of active zone [71]	Phosphorylation	-	↑	↑	-
Git1	Interaction with liprin α [71]	Phosphorylation	↓	-	↓	↑↓
Cadherin 13	Cadherin–catenin complex [72]	Expression	-	-	-	↓
Catenin β	Cadherin–catenin complex [72]	Phosphorylation	-	-	↑	↑
Catenin δ2	Cadherin–catenin complex [72]	Phosphorylation	↑	↑	-	↑
Arhgap35	Interaction with catenin δ, inactivation of RhoA [72]	Phosphorylation	-	↑	↑	-
Rock1	Activation by RhoA [72]	Expression	-	-	↓	-
Cortactin	Interaction with catenin δ [72]	Phosphorylation	-	↑	↑	↓
Shank3	Scaffold in postsynaptic density [67]	Expression	↓	-	-	-
Arhgef7 (β-PIX)	Interaction with Shank3 [67]	Phosphorylation	-	↓	-	-
Git1	Interaction with β-PIX [73]	Phosphorylation	↓	-	↓	↑↓
Cortactin	Interaction with Shank3 [67]	Phosphorylation	-	↑	↑	↓
Spectrin α	Actin–spectrin network [68]	Phosphorylation	↑	-	-	-
Spectrin β	Actin–spectrin network [68]	Phosphorylation	-	↑	↑	-
Ankyrin 2	Actin–spectrin network [68]	Phosphorylation	-	↑↓	-	↓
Alpha-adducin	Actin–spectrin network [68]	Phosphorylation	↓	↑	-	↑↓
Beta-adducin	Actin–spectrin network [68]	Phosphorylation	↑	↑↓	↑↓	↑
Gamma-adducin	Actin–spectrin network [68]	Phosphorylation	↑	↓	-	-
Band 4.1-like protein 1	Actin–spectrin network [68]	Phosphorylation	↑	↑↓	↑	-
MAP1A	Microtubule dynamics [26]	Phosphorylation	↑	↑↓	↑↓	↑
MAP1B	Microtubule dynamics [26]	Phosphorylation	↑↓	↑↓	↑↓	↑↓
MAP2	Microtubule dynamics [26]	Phosphorylation	↑↓	↑↓	↑	↓
Tau (MAPT)	Microtubule dynamics [26]	Phosphorylation	↑	↑↓	↑	↑↓
Tppp	Microtubule polymerization [74]	Phosphorylation	↑	↓	-	-
Dpysl2 (Crmp2)	Microtubule stability [75]	Phosphorylation	-	-	↑	↑
Crmp1	Microtubule stability [76]	Phosphorylation	-	↑	-	↑

↑, hyperphosphorylation or increased level of protein after a 6-month morphine withdrawal; ↓, hypophosphorylation or decreased level of protein after a 6-month morphine withdrawal; ↑↓, protein with simultaneous hyperphosphorylation at some phosphosites and hypophosphorylation at others in one experimental group; -, no change; Ctx, cortex. H, hippocampus, S, striatum, Cb, cerebellum.

### 3.4. Changes in Phosphorylation Pattern of Selected Phosphoproteins after a 6-Month Morphine Withdrawal

Because MAPs, synapsin-1 (Syn1), Bassoon and Piccolo proteins were found to be altered in all the four brain regions (Table 2, Table 3, Table 4 and Table 5, Supplementary Table S3) and were frequently present in GO enrichment analysis of altered phosphoproteins after a six-month morphine withdrawal (Supplementary Table S1), we focused on their alterations in detail. Camk2a kinase was found to be differentially phosphorylated in cortex, hippocampus and striatum (Table 2, Table 3 and Table 4).

#### 3.4.1. Changes in Phosphorylation Pattern of Selected Phosphoproteins in the Cortex

MAP1A protein was detected to be hyperphosphorylated at Ser1518, Ser2001 and Ser2005 in cortex after a six-month morphine withdrawal (Table 2). All these phosphosites are outside the microtubule-binding domain (MBD) and their function is not known [26]. MAP1B protein was hyperphosphorylated at Ser930, Ser1315, Ser1389 and Ser1393 and hypophosphorylated at Thr965 (Table 2). The phosphosites Ser1389 and Ser1393 have not been yet described, but they match Ser1388 and Ser1392 found in MAP1B purified from neonatal rat brain [77]. They are located in MTA, a microtubule assembly-helping site [78]. Phosphorylation of Ser1392 by DYRK1A kinase was found to prime the subsequent phosphorylation of Ser1388 by GSK3β kinase and both these phosphosites must be concurrently phosphorylated for regulating microtubule stability [77]. MAP2 protein was hyperphosphorylated at Thr1606, Ser1782 and Ser1784 on doubly phosphorylated phosphopeptide and hypophosphorylated at Ser1784 on triply phosphorylated phosphopeptide (Table 2). The phosphosite Thr1606 is located in the proline-rich domain and its phosphorylation is known to reduce microtubule binding by MAP2 [79,80]. By comparing the MAP2 sequence (F1MAQ5) identified in our study with rat sequence of full-length MAP2 [81], we determined that the phosphosites Ser1782 and Ser1784 are located in the repeat 4a domain. Phosphorylation of Ser1782 may act to dissociate MAP2 from microtubules [79]. Tau protein was hyperphoshorylated at Ser436, Ser440, Thr648 and Ser661 (Table 2). The detected rat tau protein (ID D4A1Q2) has a length of 686 amino acids. The largest human tau isoform has 441 amino acids and its phosphorylation sites have been widely examined [82,83,84]. By comparing the sequences of rat and human tau isoforms, rat phosphosites Ser436, Ser440, Thr648 and Ser661 were matched to human Ser191, Ser195, Thr403 and Ser416, respectively. The phosphosites Ser436 and Ser440 are located in the proline-rich domain, which is involved in binding to and bundling F-actin [85] and serves as a core tubulin-binding domain with tubulin polymerization capacity [86]. The phosphosites Thr403 and Ser416 are located in the C-terminal domain, in which site-specific phosphorylations may facilitate the process of tau assembly [87]. In the cortex, TPPP protein was hyperphosphorylated at Ser34 in the N-terminal tail after a six-month morphine withdrawal (Table 2). The function of this phosphorylation is not yet known.

Synapsin-1 was found to be hypophosphorylated at Ser430 (Table 2), a phosphosite located in domain D. Basson was hyperphosphorylated at phosphosites Ser2632 and Ser2634 (Table 2). Piccolo was hyperphosphorylated at Ser3326 and hypophosphorylated at Ser3054 (Table 2). The phosphosite Ser3054 is located in CC3 docking site engaged in scaffolding and assembly of a core complex in the cytomatrix at the active zone [88]. Camk2a kinase was hyperphosphorylated at Ser331, Ser333, Thr336 and Thr337 and hypophosphorylated at Ser330 (Table 2). All these phosphosites are located in the C-terminal domain, which mediates holoenzyme formation [89].

#### 3.4.2. Changes in Phosphorylation Pattern of Selected Phosphoproteins in the Hippocampus

MAP1A protein was hyperphosphorylated at Ser764, Ser1236 and Ser1691 and hypophosphorylated at Ser2135 in the hippocampus after a six-month morphine withdrawal (Table 3). All these phosphosites are outside the MBD domain and their function is not known [26]. MAP1B protein was hyperphosphorylated at Ser1315, Ser1371, Ser1382, Ser1465, Ser1494, Ser1772, Ser1775 and Ser1778 and hypophosphorylated at Ser14, Ser614, Ser985, Ser1239, Ser1244, Ser1254 and Ser1432 (Table 3). The phosphosite Ser14 is located in the N-terminal actin-binding domain (ABD), Ser614 in the MTB domain and cluster of phosphosites Ser985, Ser1239, Ser1244, Ser1254, Ser1315, Ser1371 and Ser1382 in MTA site. The phosphosites Ser1432, Ser1465, Ser1494, Ser1772, Ser1775 and Ser1778 are located in the sequence between the MTA site and MBD domain of the C-terminal tail [78]. The most frequent alterations in phosphorylation of MAP1B were found in a microtubule assembly-helping site MTA, suggesting that a six-month morphine withdrawal could affect microtubule assembly. MAP2 protein was hyperphosphorylated at Ser362, Ser1784, Ser1785 and Ser1793 (in doubly phosphorylated phosphopeptide) and hypophosphorylated at Ser1064, Ser1793 (in triply phosphorylated phosphopeptide), Ser1796 and Ser1797 (Table 3). The phosphosites Ser362 and Ser1064 are located in a sequence termed the end of acidic domain [81], Ser1784 in the repeat 4a domain and Ser1785, Ser1793, Ser1796 and Ser1797 in the C-terminal domain. By sequence comparison, the phosphosite Ser1793 in MAP2 (F1MAQ5) with 1825 amino acids matches Ser435 in MAP2c with 476 amino acids [90]. Phosphorylation of this phosphosite is mediated by proteinkinase A (PKA), and has an effect on the binding of 14-3-3 proteins [90], a highly abundant protein family in the brain affecting the activity and localization of substrate proteins [91].

Tau protein was hyperphosphorylated at Ser423, Thr426 (in triply phosphorylated phosphopeptide), Ser 436 (in doubly and triply phosphorylated phosphopeptides), Ser440, Ser444, Ser447 (in doubly and triply phosphorylated phosphopeptides), Ser480 and Ser661, and was hypophosphorylated at Ser45 and Thr426 (in doubly phosphorylated phosphopeptide). The phosphosite Ser45 is located in the N-terminal domain, while the phosphosites Ser423, Thr426, Ser436, Ser440 are in the proline-rich domain P1, and Ser 444, Ser447 and Ser480 in the proline-rich domain P2. The phosphosite Ser661 is located in the C-terminal domain. The most frequent alterations in phosphorylation were found in proline-rich domains, suggesting that binding of tau with actin and tubulin could have been affected by a six-month morphine withdrawal.

TPPP protein was found to be hypophosphorylated at Ser31 in the N-terminal tail (Table 3). By sequence comparison, this phosphosite in rat TPPP matched Ser32 in human TPPP/p25 [92]. This phosphosite Ser32 was shown to be phosphorylated by PKA [74] and Rock1 [93].

Synapsin-1 was hyperphosphorylated at Ser436, Ser518 and Ser682 (Table 3). The phosphosites Ser436 and Ser518 are located in the proline-rich domain D, while the phosphosite Ser682 is in domain E [94]. Basson was hyperphosphorylated at Ser1098 and Thr1100 (Table 3). Piccolo was hypophosphorylated at Ser3054 and Ser3326 (Table 3). Camk2a was hyperphosphorylated at Ser330 and Ser333 (both in doubly and triply phosphorylated peptides), while it was hypophosphorylated at Thr334 and Thr336 (in singly and triply phosphorylated peptides) (Table 3).

#### 3.4.3. Changes in Phosphorylation Pattern of Selected Phosphoproteins in the Striatum

MAP1A protein was found to be hyperphosphorylated at Ser1136 and Ser2432 and hypophosphorylated at Ser1232 in the striatum after a six-month morphine withdrawal (Table 4). All these phosphosites are outside the microtubule-binding domain and their function is not yet known [26]. MAP1B protein was hyperphosphorylated at Ser960, Ser1244 (in singly phosphorylated peptide), Ser1389, Thr1496, Ser1646, Ser1778 and Ser1781 and hypophosphorylated at Ser929, Ser930, Ser956, Ser963, Ser1239, Ser1244 (in doubly phosphorylated peptide) and Ser1494 (Table 4). The phosphosites Ser930, Ser956, Ser960 and Ser963 are located in a sequence between the C-terminal MBD domain and MTA site, the phosphosites Ser1239, Ser1244 and Ser1389 in MTA site, the phosphosites Ser1494, Thr1496, Ser1646, Ser1778 and Ser1781 in a sequence between the MTA site and the N-terminal MBD domain. The most frequent alterations in phosphorylation of Map1B were found in sequences outside the known binding domain and the function of phosphorylation of these phosphosites has not yet been described. MAP2 protein was hyperphosphorylated at phosphosites Ser1780, Ser1784 and Ser1788 in the C-terminal tail (Table 4). The function of these phosphosites is not known.

Tau protein was hyperphosphorylated only at Ser480 located in proline-rich domain P2 and at Tyr639 located in C-terminal domain (Table 4); these phosphosites match Ser235 and Tyr394 in human tau, respectively. The phosphosite Ser235, together with Ser202, Thr205 and Thr231 in human tau are phosphorylated by CDK2/CycA3 kinase in vitro, and when at least three out of these four positions are phosphorylated, tau loses its capacity to assemble tubulin into microtubules [95]. In our study, the other three phosphosites matched to Ser202, Thr205 and Thr231 in human Tau were not detected or altered (data not shown). The phosphosites Tyr394 and Ser396 in human Tau were proved to weaken the interaction between tau and microtubules and phosphorylation at Tyr394 had a more pronounced effect than phosphorylation at Tyr394 [95]. In our study, Tyr639 matched to Tyr394 in human tau was greatly hyperphosphorylated (Table 4) but phosphorylation at Ser641 matched to Ser396 in human Tau was not altered in striatum after six-month morphine abstinence suggesting only a smaller weakening of interaction between Tau and microtubules.

Synapsin-1 was found to be hyperphosphorylated at Ser425, Ser508 and Ser516 (Table 4); all these phosphosites are located in domain D [94]. Bassoon was hyperphosphorylated at Ser1220 and Ser2842 and Piccolo was hypophorylated at Ser66 and Trh2103 (Table 4). The phosphosite Thr2103 in Piccolo is located in the docking site for Daam1, which is involved in actin cytoskeleton dynamics [88]. Camk2a was hypophosphorylated at Ser330 (Table 4).

#### 3.4.4. Changes in Phosphorylation Pattern of Selected Phosphoproteins in the Cerebellum

MAP1A protein was hyperphosphorylated at Ser1860 in the cerebellum after a six-month morphine withdrawal (Table 5). This phosphosite is outside the microtubule-binding domain and its function is not known [26]. Map1B protein was hyperphosphorylated at Ser825 (in doubly phosphorylated peptide), Ser1254, Ser1317 and hypophosphorylated at Ser821, Ser824 (in doubly and triply phosphorylated peptides), Ser825 (in triply phosphorylated peptide), Ser963, Ser1319, Ser1332 and Ser1393 (Table 5). The phosphosites Ser821, Ser824, Ser825 are located in MBD domain in the heavy chain near the N-terminus of MAP1A. The phosphosites Ser963, Ser1254, Ser1317, Ser1319, Ser1332 and Ser1393 are located in MTA site. These results suggest alterations in binding of MAP1B to microtubules and microtubule assembly.

MAP2 otein was hypophosphorylated at Ser724 and Tyr744 (Table 5) in the variable central region of the protein [90]; the function of these phosphosites is not yet known. Tau protein was hyperphosphorylated at Ser649 in triply phosphorylated peptide and hypophosphorylated at Ser423, and at Thr426 and Ser649 in doubly phosphorylated peptide (Table 5). The phosphosites Ser423 and Thr426 are located in the proline-rich domain P1 and the phosphosite Ser649 in the C-terminal domain, suggesting alterations in binding of tau to actin and microtubules and in tau assembly.

Synapsin-1 was hyperphosphorylated at Ser432 located in domain D and at Ser680 in domain E and hypophosphorylated at Ser430 located in domain D (Table 5). Bassoon was hyperphosphorylated at Ser1034, Ser1035 and Thr1100 and hypophosphorylated at Ser1098 and Ser1469 (Table 5). The phosphosite Ser1469 is located in the docking site for dynein light chains (DLCs) involved in vesicle trafficking [88]. Piccolo was hyperphosphorylated at Ser2337, Ser2343, Ser3320 and Ser3326 and hypophosphorylated at Ser63 (Table 5). The phosphosites Ser2337 and 2343 are located in the docking site for GTPase-activating protein Git1 [88], which was also differentially phosphorylated after a six-month morphine withdrawal. It was hyperphosphorylated at Ser379 and Thr383 and hypophosphorylated at Ser376 (Supplementary Table S3). By comparing the sequence of rat Git1 (A0A0G2K527) with human Git1 [96], the phosphosites Ser376, Ser379 and Thr383 in rat Git1 were found to match Ser394, Ser397 and Thr401 in human Git1. These phosphosites are located in a synaptic localization domain (SLD), suggesting that the function of Git1 in synapse formation is regulated by phosphorylation [96]. Alterations in phosphorylation of Git1 in SLD domain may suggest changes in synaptic activity.

## 4. Discussion

The present study followed from previous work in which alterations in protein expression and phosphorylation were assessed by 2-D electrophoresis and label-free quantification in selected brain regions of rats three months after cessation of chronic morphine treatment [46]. Here, we observed that cytoskeletal proteins (actin and tubulin) and their binding partners (Tppp, Dpysl2, F-actin capping protein β) were differentially expressed or phosphorylated mainly in the cortex and hippocampus. The tubulin polymerization promoting protein is specifically expressed in oligodendrocytes [29], and has two cellular functions. It promotes microtubule polymerization and regulates HDAC6 activity [93]. Tppp dynamically colocalizes with microtubules and induces microtubule bundling and stabilization followed by increased acetylation of microtubules [97]. The phosphorylation of human Tppp at Ser32, corresponding to Ser31 in rat Tppp, mediated by Rock kinase contributes to inhibition of its binding to HDAC6, subsequent increase in HDAC6 activity and tubulin deacetylation [93]. While the level of Tppp protein was downregulated following a three-month morphine withdrawal in the cortex and hippocampus and hypophosphorylated in the striatum [46], a six-month morphine withdrawal resulted in hyperphosphorylation of Tppp at Ser34 in the cortex and hypophosphorylation at Ser31 in the hippocampus, suggesting that, at least in rat hippocampus, regulation of tubulin acetylation via Tppp expression or phosphorylation at Ser31 might occur over the course of several months after morphine withdrawal.

Dihydropyrimidinase-related protein 2 (Dpysl2) is another protein which was found to be differentially phosphorylated after a three- as well as six-month morphine withdrawal. Whereas this protein was hyperphosphorylated in the cortex and hypophosphorylated in the hippocampus after a three-month morphine withdrawal [46], it was hyperphosphorylated at Ser537 in the striatum and at Ser542 in the cerebellum after a six-month morphine withdrawal. The other dihydropyrimidinase-related proteins, Crmp1 (collapsin response mediator protein 1) and Dpysl3, were also differentially phosphorylated after a six-month morphine withdrawal. The majority of differentially phosphorylated sites on dihydropyrimidinase-related proteins were located in the C-terminal region, and only one differentially phosphorylated site in Crmp1 was located in the N-terminal region. Whereas the N-terminal dihydropyrimidinase-like domain appears to promote microtubule assembly, the C-terminal region of Crmp1 and Crmp2 (Dpysl2) is sufficient to stabilize the microtubules [76]. The phosphorylation of Crmps at its C-terminal domains causes microtubule destabilization, while inhibition of the C-terminal phosphorylation has a stabilizing effect [98]. The residues Thr509, Thr514 and Ser518 at the C-terminus of collapsin response mediator proteins are phosphorylated by GSK-3β [76]. In the present study, differential phosphorylation patterns at these residues in Dpysl3 (Crmp4) were observed in all four brain regions under scrutiny. Hyperphosphorylation at Thr509 and hypophosphorylation at Ser518 in Dpysl3 were found in the hippocampus and striatum, respectively. The residues Thr509 and Thr514 were simultaneously hyperphosphorylated in the cortex and hypophosphorylated in the cerebellum, indicating an opposing trend in the binding of Dpysl3 to microtubules in the cortex and cerebellum. Taken together, collapsin response mediator proteins are other microtubule-binding phosphoproteins that were affected by protracted morphine withdrawal.

Morphine belongs to addictive opioid pain relievers that are often used in medical care and have a high potential for abuse. Drug abuse is a relapsing brain disease characterized by the adaptations within the mesolimbic reward system and associated neural circuits that may persist a long time after cessation of drug intake [99]. It was demonstrated that morphine-abstinent mice develop low sociability and despair-like behavior detectable up to four weeks after discontinuation of chronic drug exposure [100]. In the cocaine self-administration model of relapse liability, many protein alterations occurring during cocaine self-administration returned to normal levels between 1 and 100 d of abstinence, but some remained altered even after 100 d. On the other hand, some proteins which were not affected during cocaine self-administration altered during the abstinence period. Differentially expressed proteins during the abstinence period may contribute to specific functions related to relapse liability [101]. In that report, proteins associated with synaptic plasticity were altered in the prefrontal cortex, suggesting the importance of synaptic communication in withdrawal-associated behavior. Cocaine administration induced changes in the level of Dpysl2 and the following drug withdrawal was associated with changed expression of SNAP-25, a component of the SNARE complex, and dynamin-1 located in the postsynaptic density [101]. This is consistent with our current results suggesting that alterations in the proteome profiles of synaptic proteins may contribute to molecular neuroadaptations associated with chronic drug exposure and long-term drug abstinence.

Synapsin-1 is one of crucial proteins whose phosphorylation occurs commonly during withdrawal from different drugs. This protein was differentially phosphorylated at Ser9, Ser62 and Ser67 in the NA two hours after cocaine self-administration and phosphorylation at Ser9 was still elevated after 22 h [102]. Phosphorylation at Ser603 of synapsin-1 was increased in the mouse NA after chronic nicotine administration and decreased 24 h after drug cessation [103]. Synapsin acts as a key protein for maintaining SVs within the reserve pool, which is a large SV cluster distal to the active zone. The reserve pool serves as a store that replenishes SVs into a readily-releasable pool following exocytosis of neurotransmitters [104]. The mechanism of maintaining SVs within the reserve pool by synapsin is unclear. The first hypothesis relies on the involvement of synapsin in cross-linking of SVs, thereby anchoring SVs to each other. The cross-linking of SVs follows dimerization and tetramerization of synapsin mediated by its conserved domain C. The second hypothesis relies on creating a liquid phase that allows SVs to float within a synapsin droplet. Such formation of liquid condensate is mediated by the variable IDR domains at the C-terminal end of the molecule [104]. It is hard to imagine that such process depends on the level of expression of these IDR domains and it should be mediated by posttranslational modifications, including phosphorylation. Interestingly, phosphorylation of synapsin-1 by CaMKII caused a disassembling of the liquid phase of synapsin [105]. In our present study, the phosphorylation of synapsin induced by a six-month morphine withdrawal occurred in phosphorylation sites in domains D and E, which are located in IDR domains at the C-terminal end of synapsin (Table 2, Table 3, Table 4 and Table 5). Although these phosphorylation sites are of unknown functions and the protein kinase phosphorylating them is not known as well, they might be proposed as phosphorylation sites of CaMKII in the formation and disassembly of the liquid phase of synapsin.

The vesicle cluster near the active zone has been suggested to be the main source for many other proteins, including Rab3, complexin, synaptobrevin (Vamp2), amphiphysin (Bin1), Rim2 (similar to Rims1), bassoon, cortactin, and tubulin [106]. In our present study, many of these proteins or their isoforms were differentially phosphorylated or expressed (Table 2, Table 3, Table 4, Table 5, Table 6 and Table 7). The vesicle cluster has been proposed to serve as a buffer for soluble accessory proteins involved in vesicle recycling and to ensure that the soluble recycling proteins are delivered upon demand during synaptic activity and thereby to support neurotransmission indirectly [106]. However, there is yet another possibile way the phosphorylation of synaptic proteins may be employed in the synaptic vesicle cycle. It might contribute to the interaction between vesicles and soluble proteins.

Some proteins from presynaptic and postsynaptic compartments engaged in cell adhesion, scaffolding, exocytosis and neurotransmitter transport may be implicated in several synaptopathies, causing neurological disorders [5]. Not only mutations and deletions in genes producing synaptic proteins, but also aberrant phosphorylation of proteins related to synaptic plasticity and cytoskeleton organization may play an important role in the pathogenesis of neurological diseases. One of the most studied phosphoprotein whose hyperphosphorylation is associated with the pathology of Alzheimer’s disease is tau [6,83,84]. Tau hyperphosphorylation alters the ability of tau to stabilize microtubules and subsequently impairs axonal transport [6]. The stabilization of microtubules was shown to improve cognitive function and axonal transport [107,108]. The other neurotoxic effects of tau hyperphosphorylation in Alzheimer’s disease include the impairment of long-term depression, NMDA receptor hypofunction, impaired neuronal hyperexcitability and reduced Fyn-induced Src family kinase activity [6]. In our study, tau was hypophosphorylated only in the cerebellum, but hyperphoshorylated in cortex, hippocampus and striatum. The greatest degree of hyperphosphorylation was detected in the hippocampus and most of the hyperphosphorylated phosphosites were located in proline-rich domains involving tubulin-binding site, the motif contributing to the regulation of tau interaction with microtubules and promoting microtubule polymerization [90], suggesting that hyperphosphorylation of tau in the hippocampus induced by long-term morphine withdrawal might affect the stabilization of microtubules associated with alterations in cognitive functions.

MAP1A, MAP1B, MAP2, collapsin response mediator proteins, α- and β-adducins, ankyrin 2, Akap12, Stxbp1, Marcks, and stathmin represent another group of differentially phosphorylated phosphoproteins whose aberrant phosphorylation is associated with neurological diseases [6]. Besides MAPs and collapsing response mediator proteins, stathmins possess microtubule-destabilizing activity, which is mediated by protein phosphorylation [109,110]. This suggests that alterations in microtubule stability can be one of the neuroadaptive mechanisms induced by long-term drug withdrawal which might be associated with changes in cognitive functions. A 30-day withdrawal from cocaine self-administration resulted in changes of Src kinase/Srcin1 signaling together with microtubule and actin remodeling followed by increased dendritic spine density and morphological restructuring of dendritic spines in NA [111]. Our results suggest the Src kinase/Srcin1 signaling as well as microtubule and actin dynamics are affected during morphine withdrawal also in the cortex, hippocampus and striatum by differential phosphorylation of Srcin1 and microtubule- and actin-associated proteins. Repeated morphine treatment elicits changes in the density of dendrites and dendritic spines in the cortex and hippocampus [112,113,114], suggesting that Src kinase/Srcin1 signaling and microtubule/actin dynamics should be a common mechanism affecting the morphology of dendrites and dendritic spines during drug withdrawal. The morphology of dendrites and dendritic spines are also regulated by Rho and Ras family of GTPases [115], as well as by some regulators of GTPases such as SynGAP, ArhGEF7, ArhGAP35 [115,116]. Dendrites and dendritic spines have been recognized to be critical for synaptic plasticity related to cognitive processes such as learning and memory. Reward learning is encoded by dendritic spine changes from the first drug exposure to relapse even long into the withdrawal period [111].

Phosphoproteomic analysis revealed the phosphosite Ser331 in CaMKII whose phosphorylation was associated with inhibition of CaMKII activity and memory extinction in the amygdala from rats self-administered with cocaine for ten days [117]. In our study, the phosphosite Ser331 in CaMKII was hyperphoshorylated in the cortex after morphine withdrawal, suggesting memory extinction in cortex associated with opioid-related reward memories [118]. Because the consequences of morphine use are long-lasting, even many months after the cessation of drug administration, it is highly desirable to find novel strategies that could reverse cellular processes leading to drug relapse. Our results suggest that therapeutic agents affecting the phosphorylation state of synaptic proteins and improving the formation of the reserve pool and morphology of dendritic spines might be considered as potential candidates for restoring synaptic function and thus reversing drug seeking and relapse during protracted withdrawal.

## 5. Conclusions

Our results demonstrate for the first time that prolonged administration and subsequent discontinuation of morphine can cause diverse neuroadaptive changes in different regions of rat brain which are detectable even six months after cessation of drug intake. Distinct changes were observed in both protein expression and phosphorylation in the cortex, hippocampus, striatum, and cerebellum. In general, changes in protein phosphorylation were more prominent than those in protein expression. Alterations in protein expression and phosphorylation were found to be associated with synaptic plasticity and cytoskeleton organization. In all four brain regions, a six-month morphine withdrawal strongly affected the phosphorylation of proteins located in the active zone of the presynaptic nerve terminal, which is the site of synaptic vesicle exocytosis. Significant alterations were uncovered in the phosphorylation of proteins engaged in microtubule dynamics and stability, as well as in organization of the spectrin–actin network. The role of many phosphosites with altered phosphorylation is still unclear and needs to be elucidated. The observed changes of the phosphoproteomic profiles of different brain regions elicited by prolonged morphine withdrawal may likely affect cognitive functions. However, this assumption requires further investigation.

## Figures and Tables

**Table 1:** Gene Ontology (GO) biological process enrichment analysis of differentially phosphorylated and expressed proteins in four selected rat brain regions six months after withdrawal from morphine (conducted by gProfiler).

Biological Processes	Ctx	Hp	Str	Cb
Synaptic vesicle cycle	P	P	P	P
Synapse organization	E	P	-	P
Neurotransmitter transport and secretion	P,E	P	P	P
Regulation of neurotransmitter level	-	P	P	P
Synaptic transmission	E	P	-	P
Synaptic signaling	P,E	P,E	P	P
Cytoskeleton organization	P	P	P,E	-
Regulation of GTPase activity	-	P	P	E

Ctx, cortex; Hp, hippocampus; Str, striatum; Cb, cerebellum; P, GO biological processes inferred from the differences in protein phosphorylation; E, GO biological processes inferred from the differences in protein expression; -, GO enriched biological process not found.

**Table 2:** A list of differentially phosphorylated proteins associated with cytoskeletal, synaptic plasticity and GTPase regulatory activity in the cortex six months after withdrawal from morphine.

Protein ID	Gene	Protein Name	Position, Fold Change
Q5HZA7	Bin1	Bin1 protein	S265 (_1) ↑(MW)
G3V984	Bsn	Protein bassoon	S2632 (_2) ↑(2.80); S2634 (_2) ↑(2.80)
P11275	Camk2a	Ca^2+^/calmodulin-dependent protein kinase type II α	S330 (_2) ↓(-4.88); S331 (_3) ↑(2.54); S333 (_3) ↑(2.62); T336 (_3) ↑(3.40); T337 (_3) ↑(4.57)
Q7TT49	Cdc42bpb	Ser/Thr-proteine kinae MRCK β	S1695 (_1) ↓(C)
A0A0G2JTD7	Clasp1	Cytoplasmic linker-associated protein 1	S596 (_1) ↑(2.64); S1050 (_1) ↑(MW)
F1M787	Ctnnd2	Catenin δ2	T455 (_1) ↑(2.01); S515 (_2) ↑(3.64)
Q62952	Dpysl3	Dihydropyrimidinase-related protein 3	T509 (_3) ↑(2.27); T514 (_2) ↑(7.12)
D3ZMI4	Epb41l1	Band 4.1-like protein 1	S766 (_2) ↑(2.07); S767 (_2) ↑(2.07); S1337 (_1) ↓(C)
A0A0G2K1Q9	Epb41l3	Erythrocyte membrane protein band 4.1-like 3	S94 (_2) ↑(MW); S97 (_2) ↓(−2.99)
A0A0G2K527	Git1	ARF GTPase-activating protein Git1	T383 (_1) ↓(C); T383 (_2) ↓(C)
A0A0G2K5C6	Map1a	Microtubule-associated protein 1A	S1518 (_1) ↑(2.76); S2001 (_3) ↑(2.37); S2005 (_3)↑(2.37)
P15205	Map1b	Microtubule-associated protein 1B	S930 (_1) ↑(6.14); T965 (_2) ↓(−2.34); S1315 (_3) ↑(3.62); S1389 (_2) ↑(2.09); S1393 (_2) ↑(2.09)
F1MAQ5	Map2	Microtubule-associated protein 2	T1606 (_2) ↑(2.84); S1782 (_2) ↑(2.12); S1784 (_2) ↑(3.20); S1784 (_3) ↓(−3.09)
D4A1Q2	Mapt	Microtubule-associated protein tau	S436 (_2) ([2.95); S440 (_2) ↑(2.74); T648 (_3) ↑(2.00); S661 (_1) ↑(MW)
Q920Q0	Palm	Paralemmin-1	S112 (_3) ↑(2.11)
D3Z9C7	Pclo	Protein piccolo	S3054 (_1) ↓(C); S3326 (_1) ↑(2.34)
Q6IRK8	Sptan1	Spectrin α chain 1	S1029 (_1) ↑(MW)
Q9QXY2	Srcin1	Src kinase signaling inhibitor 1	S342 (_2) ↓(C)
P61265	Stx1b	Syntaxin-1B protein	S10 (_1) ↑(6.58)
D3ZU84	Stxbp5l	Syntaxin-binding protein 5-like	S723 (_3) ↑(2.12); S724 (_3) ↑(2.12); S727 (_3) ↑(2.12)
P09951	Syn1	Synapsin-1	S430 (_3) ↓(C)
D3ZQL7	Tppp	Tubulin polymerization-promoting protein	S34 (_1) ↑(6.74)

↑, protein hyperphosphorylation after a 6-month morphine withdrawal; ↓, protein hypophosphorylation after a 6-month morphine withdrawal; C, protein phosphorylation detected only in samples from control rats; MW, protein phosphorylation detected only in samples from morphine-withdrawn rats.

**Table 3:** A list of differentially phosphorylated proteins associated with cytoskeletal, synaptic plasticity and GTPase regulatory activity in the hippocampus six months after withdrawal from morphine.

Protein ID	Gene	Protein Name	Position, Fold Change
Q5QD51	Akap12	A-kinase anchor protein 1	S614 (_2) ↑(5.04); S616 (_2) ↑(5.04)
A0A0G2K6R9	Ank2	Ankyrin 2	S1730 (_3) ↑(4.85); S1731 (_3) ↑(4.85); S1734 (_3) ↑(4.85); S2243 (_2) ↑(MW); S2246 (_2) ↓(C); S2532 (_2) ↑(2.17); T2535 (_2) ↑(2.17)
D4A9G6	Arhgap33	Rho GTPase-activating protein 33	T974 (_1) ↓(C)
D4AD82	Arhgap35	Rho GTPase-activating protein 35	S1179 (_1) ↑(2.99)
A0A1B0GWY	Arhgef2	Rho guanine nucleotide exchange factor 2	S916 (_1) ↓(C)
D4A1D2	Arhgef26	Rho guanine nucleotide exchange factor 26	S390 (_1) ↑(3.70)
A0A0G2QC21	Arhgef7	Rho guanine nucleotide exchange factor 7	S228 (_1) ↓(C)
Q5HZA7	Bin1	Bin1 protein	S265 (_1) ↑(MW)
M9MMM8	Brsk2	Ser/Thr-protein kinase BRSK2	T364 (_2) ↓(C); S398 (_3) ↓(C)
G3V984	Bsn	Protein bassoon	S1098 (_2) ↑(2.48); T1100 (_3) ↑(MW)
P11275	Camk2a	Ca^2+^/calmodulin-dependent protein kinase type II subunit α	S330 (_2) ↑(2.81); S330 (_3) ↑(2.25); S333 (_2) ↑(10.48); S333 (_3) ↑(3.44); T334 (_3) ↓(−2.22); T336 (_1) ↓(C); T336 (_3) ↓(−4.38)
P35565	Canx	Calnexin	S563 (_1) ↑(2.14)
Q7TT49	Cdc42bpb	Ser/Thr-proteine kinae MRCK β	S1692 (_2) ↓(−3.11); S1695 (_2) ↓(−3.11)
Q62950	Crmp1	Dihydropyrimidinase-related protein 1	S8 (_1) ↑(2.65)
F1M787	Ctnnd2	Catenin δ2	S515 (_2) ↑(4.35)
A0A1B0GWS4	Cttn	Src substrate cortactin	S125 (_2) ↑(2.41); S125 (_3) ↑(2.04); S135 (_3) ↑(2.14)
A0A0G2K7F5	Dlg4	Disks large homolog 4	S413 (_2) ↓(C)
G3V849	Dlgap1	Discs, large homolog-associated protein 1	S421 (_2) ↑(2.33)
A0A0G2JUI3	Dlgap2	Discs, large homolog-associated protein 2	S947 (_2) ↑(MW)
A0A0G2JX56	Dnajc5	DnaJ (Hsp40) homolog, subfamily C, member 5	S8 (_1) ↑(MW)
D4A0I5	Dnajc6	DnaJ (Hsp40) homolog, subfamily C, member 6	Y750 (_1) ↓(C)
Q62952	Dpysl3	Dihydropyrimidinase-related protein 3	T507 (_2) ↓(C); T509 (_2) ↑(MW)
D3ZMI4	Epb41l1	Band 4.1-like protein 1	T489 (_1) ↑(2.46); S544 (_2) ↑(2.09); S544 (_3) ↑(10.71); S546 (_2) ↑(2.09); T1252 (_1) ↓(C); S1320 (_2) ↓(−3.09); S1322 (_2) ↓(−3.09); T1324 (_2) ↓(−9.45)
A0A0G2K1Q9	Epb41l3	Erythrocyte membrane protein band 4.1-like 3	S91 (_3) ↑(2.44)
A0A0G2K5C6	Map1a	Microtubule-associated protein 1A	S764 (_1) ↑(MW); S1236 (_2) ↑(MW); S1691 (_1) ↑(2.56); S2135 (_1) ↓(-2.55)
P15205	P15205	Microtubule-associated protein 1B	S14 (_1) ↓(C); S614 (_1) ↓(-4.06); S985 (_1) ↓(C); S1239 (_2) ↓(C); S1244 (_2) ↓(C); S1254 (_2) ↓(−6.20); S1315 (_3) ↑(2.46); S1371 (_1) ↑(2.39); S1382 (_3) ↑(2.05); S1432 (_1) ↓(C); S1465 (_1) ↑(3.69); S1494 (_1) ↑(4.52); S1772 (_3) ↑(2.30); S1775 (_3) ↑(2.30); S1778 (_3) ↑(2.30)
F1MAQ5	Map2	Microtubule-associated protein 2	S362 (_2) ↑(2.64); S1064 (_1) ↓(−2.51); S1784 (_3)↑(2.10); S1785 (_3) ↑(2.76); S1793 (_2) ↑(MW); 1793 (_3) ↓(C); S1796 (_3) ↓(C); S1797 (_3) ↓(C)
D4A1Q2	Mapt	Microtubule-associated protein tau	S45 (_2) ↓(C); S423 (_3) ↑(2.15); T426 (_1) ↓(-2.68); T426 (_3) ↑[2.15]; S436 (_2) ↑[2.53]; S436 (_3) ↑(3.39); S440 (_2) ↑(2.42); S444 (_3) ↑(3.39); S447 (_1) ↑(2.39); S447 (_3) ↑(3.39); S480 (_2) ↑(2.04); S661 (_1) ↑(MW)
Q920Q0	Palm	Paralemmin-1	T141 (_2) ↑(3.52); T145 (_2) ↑(3.52)
D3Z9C7	Pclo	Protein piccolo	S3054 (_1) ↓(−3.58); S3326 (_1) ↓(−2.52)
D3ZZ81	Ppfia1	PTPRF-interacting protein alpha 1	S239 (_2) ↓(−2.17); S242 (_2) ↓(−2.17)
P63012	Rab3a	Ras-related protein Rab-3A	S188 (_1) ↓(C)
D3ZKQ4	Rabl6	RAB, member RAS oncogene family-like 6	S474 (_2) ↑(5.34)
Q5FVT1	Ralbp1	RalA-binding protein 1	S48 (_2) ↓(C); S62 (_2) ↓(C)
M0R920	Ranbp3	Ran-binding protein 3	S409 (_1) ↑(MW)
F1LM43	Rasgrf1	Ras-specific guanine nucleotide releasing factor 1	S747 (_1) ↑(2.02)
O08773	Rgs14	Regulator of G-protein signaling 14	S286 (_1) ↓(−4.28)
A0A0G2KAV8	Rims1	Regulating synaptic membrane exocytosis protein 1	S409 (_1) ↑(2.33)
A0A0G2K8W9	Sptbn1	Spectrin β chain	S2122 (_2) ↑(2.86); S2132 (_2) ↑(2.86); S2155 (_3) ↑(3.20)
Q9QXY2	Srcin1	Src kinase signaling inhibitor 1	S342 (_2) ↑(MW); S357 (_2) ↑(MW)
D3ZU84	Stxbp5l	Syntaxin-binding protein 5-like	S503 (_1) ↑(MW)
Q02563	Sv2a	Synaptic vesicle glycoprotein 2A	S127 (_1) ↓(−11.11)
P09951	Syn1	Synapsin-1	S436 (_3) ↑(2.15); S518 (_1) ↑(MW); S682 (_2) ↑(MW)
D3ZCL8	Syngap1	Ras/Rap GTPase-activating protein SynGAP	S1103 (_2) ↑(2.45)
P97610	Syt12	Synaptotagmin-12	T103 (_1) ↓(C)
D3ZQL7	Tppp	Tubulin polymerization-promoting protein	S31 (_1) ↓(−4.52)

↑, protein hyperphosphorylation after a 6-month morphine withdrawal; ↓, protein hypophosphorylation after a 6-month morphine withdrawal; C, protein phosphorylation detected only in samples from control rats; MW, protein phosphorylation detected only in samples from morphine-withdrawn rats.

**Table 4:** A list of differentially phosphorylated proteins associated with cytoskeletal, synaptic plasticity and GTPase regulatory activity in the striatum six months after withdrawal from morphine.

Protein ID	Gene	Protein Name	Position, Fold Change
F1M2D4	Arhgap23	Rho GTPase-activating protein 23	T560 (_2) ↑(MW)
D4A987	Arhgap31	Cdc42 GTPase-activating protein	S455 (_2) ↓(-2.11); S459 (_2) ↓(−2.11)
D4AD82	Arhgap35	Rho GTPase-activating protein 35	Y1105 (_1) ↑(2.30)
Q6TUE6	Arhgap5	Rho GTPase-activating protein 5	T1171 (_2) ↓(C)
G3V984	Bsn	Protein bassoon	S1220 (_1) ↑(MW); S2842 (_1) ↑(MW)
P11275	Camk2a	Ca^2+^/calmodulin-dependent protein kinase type II subunit α	S330 (_2) ↓(−2.83)
Q7TT49	Cdc42bpb	Ser/Thr-proteine kinae MRCK β	S1692 (_1) ↑(MW)
F1M787	Ctnnd2	Catenin δ2	S515 (_2) ↓(−2.41)
A0A1B0GWS4	Cttn	Src substrate cortactin	S125 (_2) ↑(6.53)
A0A0G2K7F5	Dlg4	Disks large homolog 4	S414 (_2) ↑(2.17); S417 (_2) ↑(2.17)
A0A0G2JUI3	Dlgap2	Discs, large homolog-associated protein 2	S557 (_2) ↑(2.33)
A0A0G2JX56	Dnajc5	DnaJ (Hsp40) homolog, subfamily C, member 5	S10 (_2) ↑(2.68)
P47942	Dpysl2	Dihydropyrimidinase-related protein 2	S537 (_1) ↑(2.12)
Q62952	Dpysl3	Dihydropyrimidinase-related protein 3	T518 (_2) ↓(−3.10)
D3ZMI4	Epb41l1	Band 4.1-like protein 1	S544 (_3) ↑(2.30); S546 (_3) ↑(2.30); T550 (_3) ↑(2.30); T1324 (_2) ↑(2.53)
A0A0G2K1Q9	Epb41l3	Erythrocyte membrane protein band 4.1-like 3	T540 (_2) ↑(MW)
A0A0G2K527	Git1	ARF GTPase-activating protein Git1	S379 (_3) ↓(C); T383 (_3) ↓(C)
A0A0G2K5C6	Map1a	Microtubule-associated protein 1A	S1136 (_2) ↑(MW); S1232 (_3) ↓)C); S2432 (_1) ↑(MW)
P15205	Map1b	Microtubule-associated protein 1B	S929 (_2) ↓(-2.52); S930 (_2) ↓(-2.52); S956 (_2) ↓(−2.72); S960 (_2) ↑(MW); S963 (_3) ↓(C); S1239 (_2) ↓(C); S1244 (_1) ↑(MW); S1244 (_2) ↓(C); S1389 (_1) ↑(MW); S1494 (_1) ↓(−2.69); T1496 (_1) ↑(6.78); S1646 (_1) ↑(2.12); S1778 (_2) ↑(2.01); S1781 (_3) ↑(2.05)
F1MAQ5	Map2	Microtubule-associated protein 2	S1780 (_3) ↑(6.15); S1784 (_3) ↑(2.32); S1788 (_3) ↑(6.15)
D4A1Q2	Mapt	Microtubule-associated protein tau	S480 (_2) ↑(3.75); Y639 (_3) ↑(MW)
Q920Q0	Palm	Paralemmin-1	S124 (_1) ↑(MW); T361 (_2) ↓(C); S365 (_2) ↓(C)
D3Z9C7	Pclo	Protein piccolo	S66 (_1) ↓(−2.45); T2103 (_1) ↓(−2.06)
F1LSE6	Ppfia3	Liprin α3	T678 (_1) ↓(C)
P63012	Rab3a	Ras-related protein Rab-3A	S190 (_1) ↑(MW)
F1M386	Rapgef2	Rap guanine nucleotide exchange factor 2	T1118 (_2) ↑(MW)
A0A0G2KAV8	Rims1	Regulating synaptic membrane exocytosis protein 1	S637 (_2) ↑(2.06); S640 (_2) ↑(2.06)
A0A0G2K8W9	Sptbn1	Spectrin β chain	S2155 (_2) ↑(26.05)
Q9QXY2	Srcin1	Src kinase signaling inhibitor 1	T658 (_2) ↑(MW)
Q02563	Sv2a	Synaptic vesicle glycoprotein 2A	S80 (_2) ↑(MW); S81 (_2) ↑(MW); S127 (_1) ↑(2.24)
P09951	Syn1	Synapsin-1	S425 (_1) ↑(2.39); S508 (_1) ↑(2.22); S516 (_1) ↑(MW)
D3ZZQ0	Tnik	Similar to Traf2 and NCK interacting kinase, splice variant 4	S335 (_2) ↑(MW); S766 (_1) ↓(C)

↑, protein hyperphosphorylation after a 6-month morphine withdrawal; ↓, protein hypophosphorylation after a 6-month morphine withdrawal; C, protein phosphorylation detected only in samples from control rats; MW, protein phosphorylation detected only in samples from morphine-withdrawn rats.

**Table 5:** A list of differentially phosphorylated proteins associated with cytoskeletal, synaptic plasticity and GTPase regulatory activity in the cerebellum six months after withdrawal from morphine.

Protein ID	Gene	Protein Name	Position, Fold Change
Q5QD51	Akap12	A-kinase anchor protein 12	S273 (_2) ↓(C)
A0A0G2K6R9	Ank2	Ankyrin 2	S2698 (_2) ↓(C)
Q5HZA7	Bin1	Bin1 protein	S265 (_1) ↑(MW)
G3V984	Bsn	Protein bassoon	S1034 (_2) ↑(3.26); S1035 (_2) ↑(3.26); S1098 (_2) ↓(−2.06); T1100 (_3) ↑(MW); S1469 (_2) ↓(C)
P35565	Canx	Calnexin	S582 (_1) ↓(−45.95)
Q62950	Crmp1	Dihydropyrimidinase-related protein 1	S566 (_2) ↑(2.64); S570 (_2) ↑(2.64)
A0A0G2JT93	Ctnnb1	Catenin β1	T556 (_1) ↑(2.06)
F1M787	Ctnnd2	Catenin δ2	S517 (_1) ↑(2.29)
A0A1B0GWS4	Cttn	Src substrate cortactin	S123 (_2) ↓(−4.09); S135 (_3) ↓(C)
D4A0I5	Dnajc6	DnaJ (Hsp40) homolog, subfamily C, member 6	S564 (_2) ↑(2.40)
P47942	Dpysl2	Dihydropyrimidinase-related protein 2	S542 (_1) ↑(MW)
Q62952	Dpysl3	Dihydropyrimidinase-related protein 3	Y499 (_3) ↓(−2.30); T509 (_3) ↓(−2.21); T514 (_2) ↓(C)
A0A0G2K1Q9	Epb41l3	Erythrocyte membrane protein band 4.1-like 3	S97 (_3) ↑(MW)
A0A0G2K527	Git1	ARF GTPase-activating protein Git1	S376 (_2) ↓(C); S379 (_3) ↑(MW); T383 (_3) ↑(MW)
A0A0G2K5C6	Map1a	Microtubule-associated protein 1A	S1860 (_1) ↑(7.34)
P15205	Map1b	Microtubule-associated protein 1B	S821 (_3) ↓(−2.08); S824 (_2) ↓(−2.28); S824 (_3) ↓(−2.08); S825 (_2) ↑(−2.28); S825 (_3) ↓(−2.08); S963 (_3) ↓(C); S1254 (_3) ↑(MW); S1317 (_3) ↑(MW); S1319 (_3) ↓(C); S1332 (_2) ↓(C); S1393 (_1) ↓(C)
F1MAQ5	Map2	Microtubule-associated protein 2	S724 (_2) ↓(C); Y744 (_2) ↓(C)
D4A1Q2	Mapt	Microtubule-associated protein tau	S423 (_3) ↓(C); T426 (_3) ↓(C);S649 (_2) ↓(−5.59); S649 (_3) ↑(3.33)
Q920Q0	Palm	Paralemmin-1	S244 (_2) ↑(MW); T363 (_2) ↓(C); T367 (_2) ↑(MW])
D3Z9C7	Pclo	Protein piccolo	S63 (_2) ↓(C); S2337 (_2) ↑(2.56); S2343 (_2) ↑(2.56); S3320 (_2) ↑(2.35); S3326 (_2) ↑(2.35)
D3ZKH6	Rabgap1l	Rab GTPase-activating protein 1-like	S490 (_1) ↑(MW)
D3ZKQ4	Rabl6	RAB, member RAS oncogene family-like 6	T606 (_2) ↓(C); S650 (_2) ↑(MW)
F1M386	Rapgef2	Rap guanine nucleotide exchange factor 2	S1115 (_2) ↑(MW)
P61265	Stx1b	Syntaxin-1B	S109 (_1) ↓(−2.56)
P61765	Stxbp1	Syntaxin-binding protein 1	S594 (_1) ↑(2.66)
Q02563	Sv2a	Synaptic vesicle glycoprotein 2A	S80 (_3) ↓(C); S81 (_3) ↓(C); T84 (_3) ↓(C); S127 (_1) ↑(2.70)
P09951	Syn1	Synapsin-1	S430 (_3) ↓(C); S432 (_3) ↑(2.25); S680 (_2) ↑(2.16)
P97610	Syt12	Synaptotagmin-12	S93 (_2) ↓(−2.25); S97 (_2) ↓(−2.25); T103 (_1) ↓(C)
G3V6M3	Syt2	Synaptotagmin-2	T125 (_1) ↑(299.42)

↑, protein hyperphosphorylation after a 6-month morphine withdrawal; ↓, protein hypophosphorylation after a 6-month morphine withdrawal; C, protein phosphorylation detected only in samples from control rats; MW, protein phosphorylation detected only in samples from morphine-withdrawn rats.

**Table 6:** A list of differentially expressed proteins associated with cytoskeletal, synaptic plasticity and regulation of GTPase activity in selected brain regions six months after withdrawal of morphine.

Brain Region	Protein ID	Gene	Proteine Name	Alteration
Cortex	A0A0G3JSM8	Cdc42	Cell division control protein 42 homolog	↑ (MW)
	P23385; G3V7U1	Grm1	Metabotropic glutamate receptor 1	↓ (−2.31)
	P15387; A0A0H2UI34	Kcnb1	Potassium voltage-gated channel subfamily B member1	↓ (−2.26)
	D4A7P2	Lrrtm2	Leucine-rich repeat transmembrane	↑(2.43)
	Q99P74	Rab27b	Ras-related protein Rab-27B	↑ (MW)
	P35280	Rab8a	Ras-related protein Rab-8A	↑ (2.34)
	D3ZWS0	Scrib	Scribble planar cell polarity protein	↓ (C)
	A0A0U1RRP5	Shank3	SH3 and multiple ankyrin repeat domains protein 3	↓ (C)
	Q9ET50	Stau1	Staufen double-stranded RNA-binding protein 1	↑ (2.08)
Hippocampus	F1LLX6	Cadps	Calcium-dependent secretion activator 1	↑ (MW)
	P23385;G3V7U1	Grm1	Metabotropic glutamate receptor 1	↑ (2.16)
	P59824; 0A096MJW6	Il1rapl1	Interleukin-1 receptor accessory protein-like 1	↓ (−2.03)
	P35280	Rab8a	Ras-related protein Rab-8A	↓ (C)
Striatum	F1LMV6	Dsp	Desmoplakin	↑ (MW)
	Q99PS8	Hrg	Histidine-rich glycoprotein	↓ (−2.04)
	A0A0G2K2Z2	Inpp5k	Inositol polyphosphate-5-phosphatase K	↑ (MW)
	D3ZKG5	Parvb	Parvin, beta	↓ (2.01)
	Q63644	Rock1	Rho-associated protein kinase 1	↓ (C)
	P34901	Sdc4	Syndecan-4	↑ (2.20)
	F1MA97	Thsd7a	Thrombospondin type 1 domain-containing 7A	↑ (2.19)
	Q4QQV0	Tubb6	Tubulin beta chain	↑ (2.10)
Cerebellum	Q5FVC2;A0A1B0GWY5	Arhgef2	Rho guanine nucleotide exchange factor 2	↓ (−2.38)
	Q5PQP4	Cdc42ep2	Cdc42 effector protein 2	↓ (C)
	F1M7X3	Cdh13	Cadherin 13	↓ (C)
	G3V6F4; P56558	Ogt	O-linked N-acetylglucosamine transferase	↑ (MW)
	Q3B7V5	Rab2b	Rab2b, member RAS oncogene family	↓ (C)
	D3ZZP2	Rab39a	Rab39, member RAS oncogene family	↓ (C)
	P70550	Rab8b	Ras-related protein Rab-8B	↑ (MW)
	A0A0G2JU11;F1M386	Rapgef2	Rap guanine nucleotide exchange factor 2	↓ (−2.41)
	P28818; A0A0G2JZ23	Rasgrf1	Ras-specific guanine nucleotide-releasing factor 1	↓ (−2.21)
	Q5BJU0	Rras2	Ras-related 2	↓ (C)

↑, protein hyperphosphorylation after a 6-month morphine withdrawal; ↓, protein hypophosphorylation after a 6-month morphine withdrawal; C, protein phosphorylation detected only in samples from control rats; MW, protein phosphorylation detected only in samples from morphine-withdrawn rats.

## Data Availability

Data is contained within the article.

## Supplementary Materials

The following are available online at https://www.mdpi.com/article/10.3390/life11070683/s1, Table S1: The enrichment analysis of differentially phosphorylated proteins in four regions of rat brain after a 6-month morphine withdrawal, Table S2: The enrichment analysis of differentially expressed proteins in four regions of rat brain after a 6-month morphine withdrawal, Table S3: A list of differentially phosphorylated proteins related to cytoskeleton, synaptic plasticity and regulation of small GTPase activity in four regions of rat brain, Table S4: A list of differentially expressed proteins related to cytoskeleton, synaptic plasticity and regulation of small GTPase activity in four regions of rat brain after.
